# One-pot gold-catalyzed synthesis of 3-silylethynyl indoles from unprotected *o*-alkynylanilines

**DOI:** 10.3762/bjoc.7.65

**Published:** 2011-05-04

**Authors:** Jonathan P Brand, Clara Chevalley, Jérôme Waser

**Affiliations:** 1 Laboratory of Catalysis and Organic Synthesis, Ecole Polytechnique Fédérale de Lausanne, EPFL SB ISIC LCSO, BCH4306, 1015 Lausanne, Switzerland

**Keywords:** alkynylation, direct functionalization, gold, hypervalent iodine, indoles

## Abstract

The Au(III)-catalyzed cyclization of 2-alkynylanilines was combined in a one-pot procedure with the Au(I)-catalyzed C3-selective direct alkynylation of indoles using the benziodoxolone reagent TIPS-EBX to give a mild, easy and straightforward entry to 2-substituted-3-alkynylindoles. The reaction can be applied to unprotected anilines, was tolerant to functional groups and easy to carry out (RT, and requires neither an inert atmosphere nor special solvents).

## Introduction

**Scheme 1:**
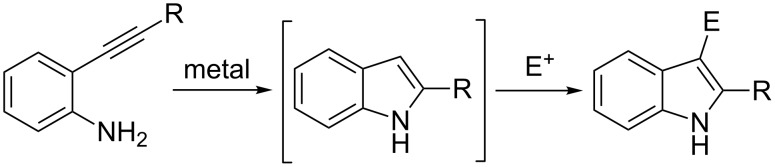
Domino cyclization–substitution reactions of 2-alkynylanilines.

Indoles are widespread in both natural products and synthetic drugs [1–2] and as a result, their synthesis and functionalization have been extensively studied [3–4]. Among the numerous syntheses of indoles, the cyclization of 2-alkynylanilines has the advantage that the resulting products, 2-substituted indoles, are easily functionalized by electrophilic aromatic substitution at position 3. Traditionally, this transformation has been achieved in two separate steps, with isolation and purification of the 3-unsubstituted indole intermediate. Domino or one-pot processes constitute a more efficient access to organic molecules, as they avoid the use of time and resource consuming work-up, and purification procedures [5–7]. When considering the importance of multi-functionalized indoles, it is therefore not surprising that the aniline cyclization–electrophilic substitution sequence has been achieved by means of several metal-catalyzed domino processes (Scheme 1) [8–10].

Among the different π-activating metals capable of promoting nucleophilic attack on acetylenes, gold has recently attracted interest from the synthetic chemistry community [11–14]. Gold catalysts have also been used in domino sequences starting from *o*-alkynylanilines. Arcadi and Marinelli reported that gold-catalyzed cyclization of 2-alkynylanilines can be followed by 1,4-addition to enones [15–16], iodination [17] or reaction with 1,3-dicarbonyl compounds [18]. Perumal recently demonstrated that aldehydes and nitroalkenes can be used as electrophilic partners [19–20]. Triple bonds can also serve as a second electrophile for the construction of tetrahydrofurans [21] and aryl-annulated[*a*]carbazoles [22]. Nakamura examined the cyclization of *N*-tosyl-*o*-alkynylanilines and observed an internal transfer of the sulfonyl group to the 3-position of the formed indoles [23–24]. Similar transformations were also achieved for the transfer of carbonyl groups, but using platinum catalysts [25–28].

To date, there are no gold- or platinum-catalyzed methods for the introduction of acetylenes as electrophiles. However, Cacchi developed a palladium-catalyzed domino sequence including cyclization of *o*-alkynyltrifluoroacetanilides and alkynylation with bromoacetylenes [29]. New methods are needed to expand the scope of this transformation and Au catalysis appears especially promising, due to its broad functional groups tolerance, which could allow the direct use of unprotected anilines.

**Scheme 2:**
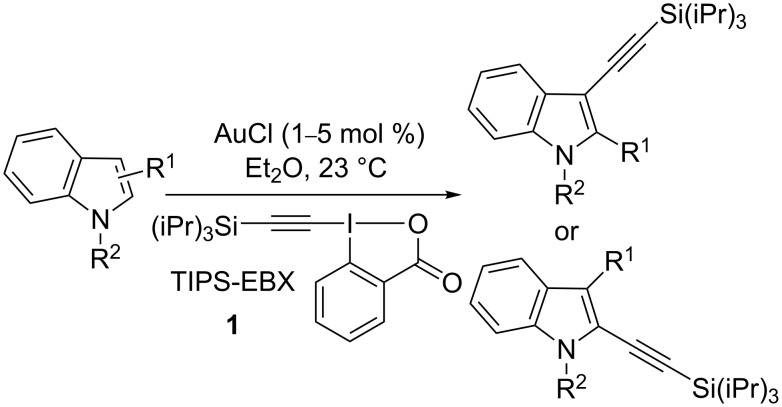
Gold-catalyzed direct alkynylation of indoles with TIPS-EBX (**1**).

Recently, the direct alkynylation of preformed heterocycles has been intensively investigated [30–34]. Most of the developed methods involve the use of haloacetylenes. In contrast, our group has focused on the use of more reactive alkynyl hypervalent iodine reagents in order to expand the scope of direct alkynylation methods under milder conditions. We recently reported a mild procedure for the C3-selective alkynylation of indoles using AuCl and the commercially available benziodoxolone TIPS-EBX (1-[(triisopropylsilyl)ethynyl]-1,2-benziodoxol-3(1*H*)-one (**1**)) (Scheme 2) [35–40]. This methodology allowed the ethynylation of a wide range of indoles, including 2-substituted indoles.

**Scheme 3:**
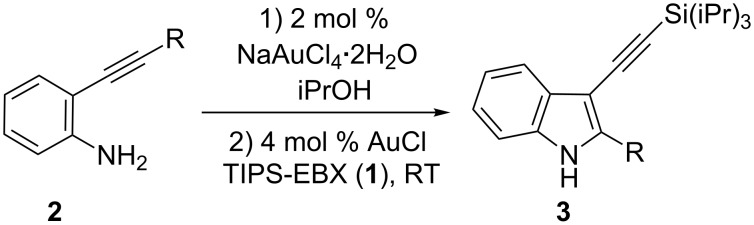
One-pot alkynylaniline cyclization/direct alkynylation.

In this letter we would like to report the one-pot combination of the cyclization of 2-alkynylanilines using NaAuCl_4_ as catalyst [15] followed by C3-alkynylation with AuCl and TIPS-EBX (**1**) (Scheme 3). This method offers an operationally simple access to 3-silylalkynyl indoles. To the best of our knowledge, this is the first example of a one-pot process combining a Au(III) and a Au(I) catalyst.

## Findings

**Scheme 4:**
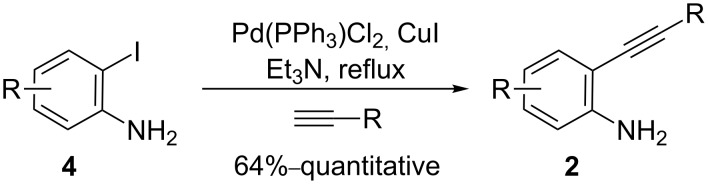
Synthesis of 2-alkynylanilines **2**.

2-Alkynylanilines **2** can be efficiently prepared from 2-iodoanilines **4** and terminal alkynes via Sonogashira reaction with Et_3_N as solvent (Scheme 4) [41–42]. The reaction was complete in less than 2 h and did not require aniline protection, solvent degassing or drying.

**Scheme 5:**
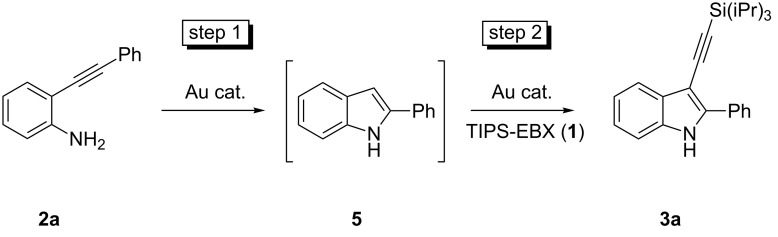
Domino cyclization–alkynylation of aniline **2a**.

Our first investigations were focused on the cyclization of 2-(phenylethynyl)aniline (**2a**) into 2-phenylindole (**5**) with AuCl as the catalyst at room temperature (Scheme 5, step 1). Since AuCl has been shown to be the best catalyst for the alkynylation reaction [35], its use would allow a domino process with a single catalyst.

Despite the fact that the use of AuCl has been reported for step 1 [19–20], in our hands the reaction was not reproducible at room temperature in a variety of solvents (EtOH, CH_3_CN, Et_2_O). A black precipitate was observed after catalyst addition, which we postulate was due to the stochastically degradation of AuCl under these conditions. NaAuCl_4_ has also been reported to be successful for the cyclization of 2-(phenylethynyl)aniline (**2a**) [15]. This catalyst was next examined in different solvents in order to maximize the chance of finding conditions suitable for both steps of the process. Aniline **2a** was fully converted into 2-phenylindole (**5**) in EtOH, iPrOH and Et_2_O after 3 h at room temperature using 2 mol % of NaAuCl_4_ and there was no problem of reproducibility. Unfortunately, NaAuCl_4_ was not an efficient catalyst for the direct alkynylation of indole, as no reaction was observed when TIPS-EBX (**1**) was added to the reaction mixture.

We then investigated the successive addition of NaAuCl_4_ and AuCl in the same pot. Interestingly, one-pot sequential processes using both Au(I) and Au(III) have not yet been reported. AuCl and TIPS-EBX (**1**) were added when full conversion of the NaAuCl_4_-catalyzed cyclization was observed. When 2 mol % of NaAuCl_4_ and 2 mol % AuCl were added, the second step was unsuccessful. However, with 2 mol % of NaAuCl_4_ and 4 mol % of AuCl, full conversion was observed after 30 h at room temperature in iPrOH (compared with 60% in EtOH and 40% in Et_2_O). A basic work-up allowed the removal of the 2-iodobenzoic acid and column chromatography afforded the product in 96% yield (average of two reactions). Unfortunately, no reaction was observed when AuCl and TIPS-EBX (**1**) were added at the beginning of the reaction.

**Scheme 6:**
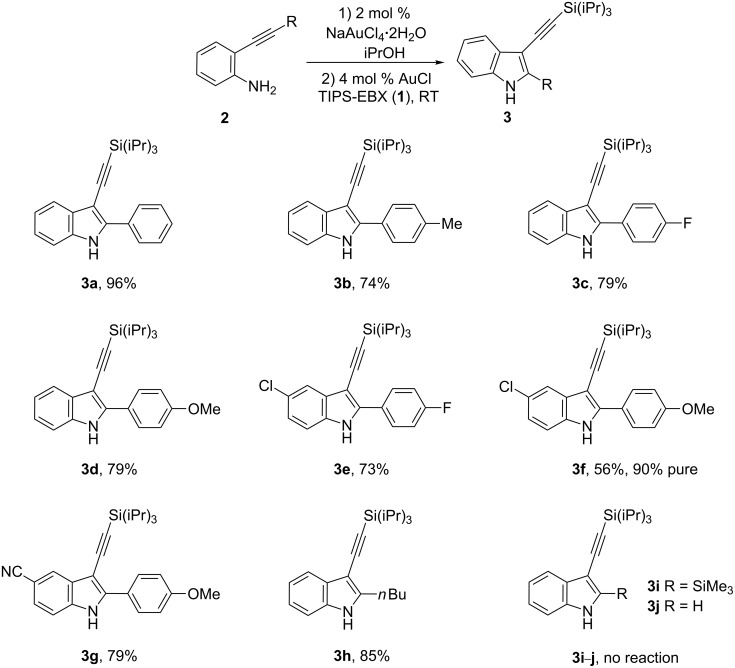
Scope of the reaction.

The scope of the reaction was then investigated (Scheme 6). Methyl- and fluoro groups were tolerated on the 2-aryl substituent to give products **3b** and **3c** in good yields. The low solubility of the indole intermediate in the synthesis of **3d** led to a low yield for the direct alkynylation step. The addition of CH_2_Cl_2_ overcame this problem. Chloro substitution in para-position of the aniline was also tolerated (**3e**, **3f**). Nevertheless, when the strongly electron-withdrawing cyano group was present, the cyclization step was too slow at room temperature. However, the use of 4 mol % of NaAuCl_4_ and a reaction temperature of 80 °C led to the formation of the desired indole, which could then be alkynylated at room temperature to give **3g**. *o*-Hexynyl aniline (**2h**) was efficiently transformed into **3h** in 85% yield. In order to access 2-silyl indoles, the synthesis of the 2-trimethylsilylacetylene substituted compound **3i** was attempted. Unfortunately, only traces of the indole intermediate were observed in this case. The reaction with 2-ethynylaniline to give (**3j**) was also unsuccessful as previously reported [16].

These first results on the direct alkynylation reaction combined in a one-pot procedure with gold-catalyzed indole ring formation are promising, and analogous strategies combining palladium-catalyzed synthesis of indoles [3] and gold-catalyzed alkynylation could also be envisaged. The next step will be to attempt a one-pot 3-steps synthesis of alkynyl indoles starting directly from iodoaniline.

In conclusion, an efficient access to 2-substituted 3-silylalkynyl indoles is reported. 2-Alkynylanilines underwent a sequential one-pot Au(III)-catalyzed annulation and Au(I)-mediated direct alkynylation. Importantly, this transformation did not require prior aniline protection and proceeded under mild conditions. This methodology represents the first example of the sequential addition of Au(III) and Au(I) catalysts for a one-pot process.

## Experimental

### General procedure for the synthesis of 2-alkynylanilines **2**

A solution of 2-iodoaniline (**4**) (1 equiv), terminal alkyne (1.2–1.3 equiv), PdCl_2_(PPh_3_)_2_ (10 mol %) and CuI (10 mol %) was heated under reflux in Et_3_N (15 mL) for 1.5–2 h under a nitrogen atmosphere. The resulting mixture was filtered through Celite^®^, washed with DCM and concentrated under vacuum. The resulting solid was purified by column chromatography.

### General procedure for the synthesis of 2-substituted 3-alkynyl indoles **3**

NaAuCl_4_ (2–4 mol %) was added to a stirred solution of 2-alkynylaniline **2** (0.40 mmol, 1 equiv) in iPrOH (3 mL) under an ambient atmosphere. The reaction was stirred at RT (80 °C for **3g**) until full conversion (3 h). TIPS-EBX (**1**) (1.2–2.4 equiv) and then AuCl (4–8 mol %) were added. The reaction was stirred until full conversion (4–30 h) and then concentrated under vacuum. Et_2_O (20 mL) was added and the organic layer was washed twice with 0.1 M NaOH (20 mL). The aqueous layers were combined and extracted with Et_2_O (20 mL). The organic layers were combined, washed successively with saturated NaHCO_3_ (20 mL) and brine (20 mL), dried with MgSO_4_ and concentrated under reduced pressure. The crude product was purified by flash chromatography.

## Supporting Information

File 1 Experimental details and spectra of new compounds.
