# Supplementary material for: One-pot gold-catalyzed synthesis of 3-silylethynyl indoles from unprotected o-alkynylanilines
Source: Beilstein J Org Chem. 2011 May 4;7:565–9. doi: 10.3762/bjoc.7.65 (PMC3107533; doi:10.3762/bjoc.7.65)
Supplement: File 1 — Experimental details and spectra of new compounds. [file Beilstein_J_Org_Chem-07-565-s001.pdf]

# Supporting Information

for

# One-pot gold-catalyzed synthesis of 3-silylethynyl indoles from unprotected *o*-alkynylanilines

Jonathan P. Brand, Clara Chevalley and Jérôme Waser*

Address: Laboratory of Catalysis and Organic Synthesis, Ecole Polytechnique Fédérale de Lausanne, EPFL SB ISIC LCSO, BCH4306, 1015 Lausanne, Switzerland

Email: Jonathan P. Brand - jonathan.brand@epfl.ch; Jérôme Waser* - jerome.waser@epfl.ch

* Corresponding author

# Experimental details and spectra of new compounds

## Table of contents

General procedures ........ S2
TIPS-EBX (1) synthesis ........ S3
2-Alkynylanilines synthesis ........ S5
Sequential annulations/Direct alkynylation ........ S10
References ........ S15
Spectra of new compounds ........ S16

## General procedures

All reactions were carried out in oven dried glassware under an atmosphere of nitrogen, unless stated otherwise. For quantitative flash chromatography technical grade solvents were used. For flash chromatography for analysis, HPLC grade solvents from Sigma-Aldrich were used. THF, $Et_2O$, $CH_3CN$, toluene, hexane and $CH_2Cl_2$ were dried by passage over activated alumina under a nitrogen atmosphere ($H_2O$ content < 10 ppm, *Karl-Fischer* titration). $NaAuCl_4$ and AuCl were purchased from Aldrich and stored in desiccators under anhydrous condition [decrease of reactivity has been observed for the catalysts on prolonged exposed to air (ca. 1 month)]. All chemicals were purchased from Acros, Aldrich, Fluka, VWR, Aplichem, Maybridge, TCI or Merck and used as received unless stated otherwise. Chromatographic purification was performed by flash chromatography on Macherey-Nagel silica 40–63, 60 Å, using the solvents indicated as eluent at 0.1–0.5 bar pressure.TLC was performed on Merck silica gel 60 $F_{254}$ TLC glass plates or aluminium plates and visualized with UV light, permanganate stain, CAN stain or anisaldehyde stain. Melting points were measured on a Büchi B-540 melting point apparatus using open glass capillaries, the data is corrected. $^1$H NMR spectra were recorded on a Bruker DPX-400 400 MHz spectrometer in $CDCl_3$, DMSO-$d_6$ or $CD_3OD$, all signals are reported in ppm with the internal chloroform signal at 7.26 ppm, the internal DMSO signal at 2.50 ppm or the internal methanol signal at 3.30 ppm as standard. The data is reported as (s = singlet, d = doublet, t = triplet, q = quadruplet, qi = quintet, m = multiplet or unresolved, br = broad signal, app = apparent, coupling constant(s) in Hz, integration, interpretation).$^{13}$C NMR spectra were recorded with $^1$H-decoupling on a Bruker DPX-400 spectrometer at 100 MHz in $CDCl_3$, DMSO-$d_6$ or $CD_3OD$, all signals are reported in ppm with the internal chloroform signal at 77.0 ppm, the internal DMSO signal at 39.5 ppm or the internal methanol signal at 49.0 ppm as standard. Infrared spectra were recorded on a JASCO FT-IR B4100 spectrophotometer with an ATR PRO410-S and a ZnSe prism and are reported in $cm^{-1}$ (w = weak, m = medium, s = strong, br = broad). Gas chromatographic and low resolution mass spectrometric measurements were performed on a Perkin-Elmer Clarus 600 gas chromatography and mass spectrometer using a Perkin-Elmer Elite fused silica column (length: 30 m, diameter: 0.32 mm) and helium as the carrier gas. High resolution mass spectrometric measurements were performed by the mass spectrometry service of ISIC at the EPFL on a MICROMASS (ESI) Q-TOF Ultima API.

## TIPS-EBX (1) synthesis

### 1-Hydroxy-1,2-benziodoxol-3(1*H*)-one (7)

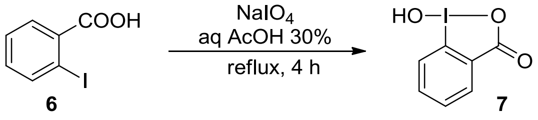


Following a reported procedure [1], $NaIO_4$ (6.7 g, 31 mmol; 1.0 equiv) and 2-iodobenzoic acid (**6**) (7.4 g, 30 mmol, 1.0 equiv) were suspended in 30% (v:v) aq. AcOH (45 mL). The mixture was vigorously stirred and heated under reflux for 4 h. The reaction mixture was then diluted with cold water (120 mL) and allowed to cool to room temperature with protection from light. After 1 h, the crude product was collected by filtration, washed on the filter with ice cold water (3 × 30 mL) and acetone (3 × 30 mL), and air dried in the dark to give pure **7** (7.3 g, 19 mmol, 92% yield) as a colorless solid. $^1$H NMR (400 MHz, $(CD_3)_2SO$) δ 8.02 (dd, *J* = 7.7, 1.4 Hz, 1 H, Ar*H*), 7.97 (m, 1 H, Ar*H*), 7.85 (dd, *J* = 8.2, 0.7 Hz, 1 H, Ar*H*), 7.71 (td, *J* = 7.6, 1.2 Hz, 1 H, Ar*H*). $^{13}$C NMR (100 MHz, $(CD_3)_2SO$) δ 167.7, 134.5, 131.5, 131.1, 130.4, 126.3, 120.4. IR ν 3083 (w), 3060 (w), 2867 (w), 2402 (w), 1601 (m), 1585 (m), 1564 (m), 1440 (m), 1338 (s), 1302 (m), 1148 (m), 1018 (w), 834 (m), 798 (w), 740 (s), 694 (s), 674 (m), 649 (m). The characterization data for compounds **7** corresponded to the reported values [1].

### Triisopropylsilyl trimethylsilylacetylene (9)

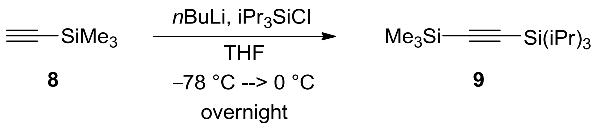


Following a reported procedure [2], *n*-butyllithium (2.5 M in hexanes, 12.0 mL, 29.9 mmol, 0.98 equiv) was added dropwise to a stirred solution of ethynyltrimethylsilane (**8**) (3.0 g, 30 mmol, 1.0 equiv) in THF (48 mL) at −78 °C. The mixture was then warmed to 0 °C and stirred for 5 min. The mixture was then re-cooled to −78 °C and chlorotriisopropylsilane (6.4 mL, 30 mmol, 1.0 equiv) added dropwise. The mixture was allowed to warm to room temperature and stirred overnight. A saturated solution of ammonium chloride (40 mL) was added, and the reaction mixture extracted with diethyl ether (2 × 60 mL). The organic layer was washed successively with water and brine, then dried over $MgSO_4$, filtered and concentrated under reduced pressure to give a colorless liquid which was further purified by Kugelrohr distillation (56–57 °C/0.25 mmHg) to yield **9** (7.16 g, 28.0 mmol, 92% yield) as a colorless liquid. $^1$H NMR (400 MHz, $CDCl_3$) δ 1.08 (m, 21 H, TIPS), 0.18 (s, 9 H, TMS). IR

ν 2959 (m), 2944 (m), 2896 (w), 2867 (m), 1464 (w), 1385 (w), 1250 (m), 996 (w), 842 (s), 764 (s), 675 (m), 660 (m). Characterization data of **16** corresponded to the literature values [2].

**1-[(Triisopropyllsilyl)ethynyl]-1,2-benziodoxol-3(1*H*)-one (TIPS-EBX, 1)**

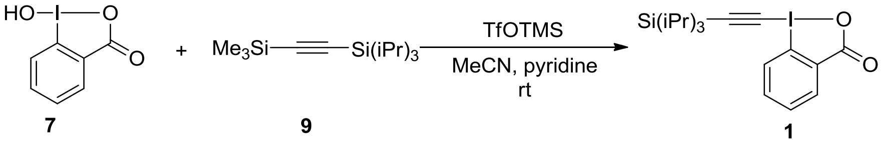


Following a reported procedure [3], 2-iodosylbenzoic acid (**7**) (21.7 g, 82.0 mmol, 1.0 equiv) was charged in oven-dried three-neck 1L flask equipped with a magnetic stirrer. After 3 vacuum/nitrogen cycles, anhydrous acetonitrile (500 mL) was added via canula and cooled to 4 °C. Trimethylsilyltriflate (16.4 mL, 90.0 mmol, 1.1 equiv) was added dropwise via a dropping funnel over 30 min (no temperature increase was observed). After 15 min, (trimethylsilyl)(triisopropylsilyl)acetylene (**9**) (23.0 g, 90.0 mmol, 1.1 equiv) was added via canula over 15 min (no temperature increase was observed). After 30 min, the suspension became an orange solution. After 10 min, pyridine (7.0 mL, 90 mmol, 1.1 equiv) was added via syringe. After 15 min, the reaction mixture was transferred to a one-neck 1L flask and concentrated under vacuum until a solid was obtained. The solid was dissolved in DCM (200 mL) and transferred to a 1 L separatory funnel. The organic solution was added, washed with 1 M HCl (200 mL) and the aqueous layer extracted with $CH_2Cl_2$ (200 mL). The organic layers were combined, washed with a saturated solution of $NaHCO_3$ (2 × 200 mL), dried over $MgSO_4$, filtered and the solvent was evaporated under reduced pressure. Recrystallization from acetonitrile (ca. 120 mL) afforded **1** (30.1 g, 70.2 mmol, 86%) as colorless crystals. Mp (dec.) 170–176 °C. $^1$H NMR (400 MHz, $CDCl_3$) δ 8.44 (m, 1 H, Ar*H*), 8.29 (m, 1 H, Ar*H*), 7.77 (m, 2 H, Ar*H*), 1.16 (m, 21 H, TIPS). $^{13}$C NMR (100 MHz, $CDCl_3$) δ 166.4, 134.6, 132.3, 131.4, 131.4, 126.1, 115.6, 114.1, 64.6, 18.4, 11.1. IR ν 2943 (m), 2865 (m), 1716 (m), 1618 (m), 1604 (s), 1584 (m), 1557 (m), 1465 (m), 1439 (w), 1349 (m), 1291 (m), 1270 (w), 1244 (m), 1140 (m), 1016 (m), 999 (m), 883 (m), 833 (m), 742 (m), 702 (s), 636 (m). Characterization data of **1** corresponded to the literature values [3].

## 2-Alkynylanilines synthesis

### 2-(Phenylethynyl)aniline (2a)

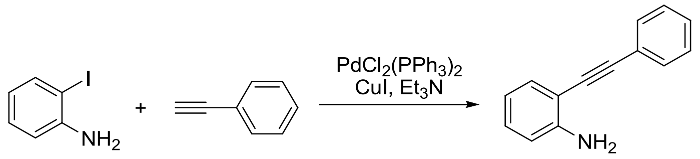


**4a** **10** **2a**

Following a slightly modified procedure [4], phenylacetylene (**10**) (1.20 ml, 11.0 mmol, 1.2 equiv) was added to a solution of 2-iodoaniline (**4a**) (2.0 g, 9.1 mmol, 1 equiv), $PdCl_2(PPh_3)_2$ (309 mg, 0.440 mmol, 0.05 equiv) and CuI (84 mg, 0.44 mmol, 0. 2 equiv) in $Et_3N$ (50 mL). The resulting suspension was stirred for 2 h under a nitrogen atmosphere at RT. The resulting mixture was filtered through Celite® and concentrated under vacuum. The resulting solid was purified by column chromatography (pentane/EtOAc 8/2) to afford **2a** (1.85 g, 9.57 mmol, quant.) as an orange solid. Mp: 85–87 °C (lit [4] 91–92 °C). Rf (pentane/EtOAc 8/2): 0.4. $^1$H NMR (400 MHz, $CDCl_3$) δ 7.54 (m, 2 H, ArH), 7.36 (m, 4 H, ArH), 7.15 (td, 1H, *J* = 7.7, 1.5 Hz, ArH), 6.73 (m, 2 H, ArH), 4.28 (s, 2 H, $NH_2$). $^{13}$C NMR (100 MHZ, $CDCl_3$) δ 147.7, 132.1, 131.4, 129.7, 128.4, 128.2, 123.3, 118.0, 114.3, 107.9, 94.7, 85.8. $^1$H NMR is consistent with reported values [4].

### 2-(*p*-Tolylethynyl)aniline (2b)

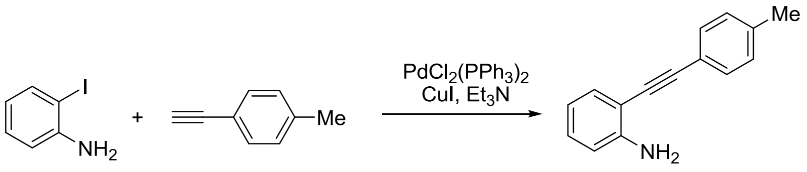


**4a** **11** **2b**

Following a slightly modified procedure [5], a solution of 2-iodoaniline (**4a**) (329 mg, 1.50 mmol, 1 equiv), 1-ethynyl-4-methylbenzene (**11**) (209 mg, 1.80 mmol, 1.2 equiv), $PdCl_2(PPh_3)_2$ (102 mg, 0.150 mmol, 0.1 equiv) and CuI (28 mg, 0.15 mmol, 0.1 equiv) were heated under reflux in $Et_3N$ (15 mL) for 2 h under a nitrogen atmosphere. The resulting mixture was filtered through Celite®, washed with DCM and concentrated under vacuum. The resulting solid was purified by column chromatography (pentane/EtOAc 95/5) to afford **2b** (276 mg, 1.33 mmol, 88%) as a yellow solid. Mp: 104–107 °C. Rf (pentane/EtOAc 95/5): 0.3. $^1$H NMR (400 MHz, $CDCl_3$) δ 7.45 (d, 2 H, *J* = 8.1 Hz, ArH), 7.38 (m, 1 H, ArH), 7.16 (m, 3 H, ArH), 6.74 (m, 2 H, ArH), 4.28 (s, 2 H, $NH_2$), 2.39 (s, 3 H, $CH_3$). $^{13}$C NMR (100 MHZ, $CDCl_3$) δ 147.7, 138.3, 132.0, 131.3, 129.5, 129.1, 120.2, 117.9, 114.2, 108.1, 94.8, 85.2, 21.5. IR ν 3475 (w), 3376 (w), 3056 (w), 3027 (w), 2919 (w), 2863 (w), 2207 (w), 1911 (w),

1611 (s), 1567 (w), 1509 (m), 1489 (m), 1456 (m), 1312 (m), 1258 (w), 1182 (w), 1157 (w), 1028 (w), 940 (w), 909 (w), 869 (w), 819 (s), 747 (s). HRMS (ESI) calcd for $C_{15}H_{14}N^+$ $[M+H]^+$ 208.1121; found 208.1125.

**2-((4-Fluorophenyl)ethynyl)aniline (2c)**

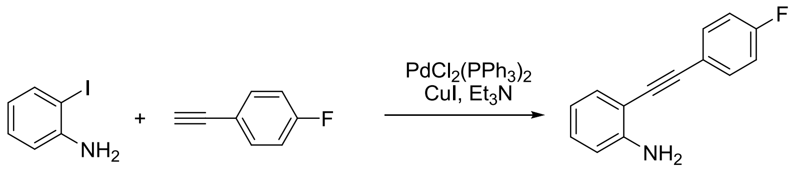


Following a slightly modified procedure [5], a solution of 2-iodoaniline (**4a**) (329 mg, 1.50 mmol, 1 equiv), 1-ethynyl-4-fluorobenzene (**12**) (216 mg, 1.80 mmol, 1.2 equiv), $PdCl_2(PPh_3)_2$ (105 mg, 0.15 mmol, 0.1 equiv) and CuI (28 mg, 0.15 mmol, 0.1 equiv) were heated under reflux in $Et_3N$ (15 mL) for 2 h under a nitrogen atmosphere. The resulting mixture was filtered through Celite®, washed with DCM and concentrated under vacuum. The resulting solid was purified by column chromatography (pentane/EtOAc 8/1) to afford **2c** (208 mg, 0.985 mmol, 66%) as a yellow solid. Mp: 97–98 °C (lit [5], 79–81 °C). Rf (pentane/EtOAc 8/1): 0.4. $^1$H NMR (400 MHz, $CDCl_3$) δ 7.50 (m, 2 H, ArH), 7.35 (d, 1 H, *J* = 8.1 Hz, ArH), 7.15 (td, 1H, *J* = 7.9, 1.5 Hz, ArH), 7.05 (t, 2 H, *J* = 8.7 Hz, ArH), 6.72 (m, 2 H, ArH), 4.25 (s, 2 H, $NH_2$). $^{13}$C NMR (100 MHZ, $CDCl_3$) δ 162.4 (d, *J* = 249 Hz), 147.7, 133.2 (d, *J* = 8 Hz), 132.0, 129.7, 119.3 (d, *J* = 3 Hz), 117.9, 115.6 (d, *J* = 22 Hz), 114.3, 107.6, 93.5, 85.5. Consistent with reported values [5].

**2-((4-Methoxyphenyl)ethynyl)aniline (2d)**

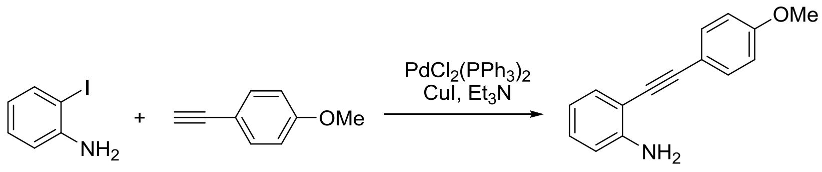


Following a slightly modified procedure [5], a solution of 2-iodoaniline (**4a**) (329 mg, 1.50 mmol, 1 equiv), 1-ethynyl-4-methoxybenzene (**13**) (258 mg, 1.95 mmol, 1.2 equiv), $PdCl_2(PPh_3)_2$ (105 mg, 0.15 mmol, 0.1 equiv) and CuI (28 mg, 0.15 mmol, 0.1 equiv) were heated under reflux in $Et_3N$ (15 mL) for 2 h under a nitrogen atmosphere. The resulting mixture was filtered through Celite®, washed with DCM and concentrated under vacuum. The resulting solid was purified by column chromatography (pentane/EtOAc 8/1) to afford **2d** (330 mg, 1.48 mmol, 98%) as a yellow solid. Mp: 109–110 °C. Rf (pentane/EtOAc 8/1): 0.3. $^1$H NMR (400 MHz, $CDCl_3$) δ 7.46 (dt, 2 H, *J* = 9.5, 2.6 Hz, ArH), 7.35 (ddd, 1 H, *J* = 7.5, 1.6, 0.6 Hz, ArH), 7.12 (ddd, 1 H, *J* = 8.1, 7.3, 1.6 Hz, ArH), 6.88 (dt, 2 H, *J* = 9.4, 2.6 Hz,

ArH), 6.71 (m, 2 H, ArH), 4.25 (m, 2 H, $NH_2$), 3.83 (s, 3 H, $CH_3$). $^{13}$C NMR (100 MHZ, $CDCl_3$) δ 159.6, 147.6, 132.9, 132.0, 129.4, 118.0, 115.4, 114.3, 114.0, 108.3, 94.6, 84.4, 55.3. Consistent with reported values [5].

**4-Chloro-2-((4-fluorophenyl)ethynyl)aniline (2e)**

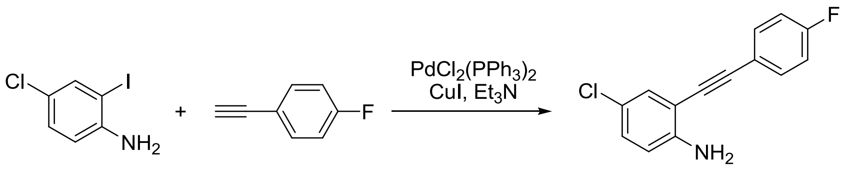


Following a slightly modified procedure [5], a solution of 4-chloro-2-iodoaniline (**4b**) (304 mg, 1.20 mmol, 1 equiv), 1-ethynyl-4-fluorobenzene (**12**) (173 mg, 1.44 mmol, 1.2 equiv), $PdCl_2(PPh_3)_2$ (84 mg, 0.12 mmol, 0.1 equiv) and CuI (23 mg, 0.12 mmol, 0.1 equiv) was heated under reflux in $Et_3N$ (12 mL) for 2 h under a nitrogen atmosphere. The resulting mixture was filtered through Celite®, washed with DCM and concentrated under vacuum. The resulting solid was purified by column chromatography (pentane/$Et_2O$ 8/2 to 6/4) to afford **2e** (283 mg, 1.15 mmol, 96%) as a yellow solid. Mp: 84–86 °C. Rf (pentane/$Et_2O$ 8/2): 0.3. $^1$H NMR (400 MHz, $CDCl_3$) δ 7.50 (m, 2 H, ArH), 7.32 (d, 1 H, $J$ = 2.4 Hz, ArH), 7.07 (m, 3 H, ArH), 6.65 (d, 1 H, $J$ = 8.7 Hz, ArH), 4.26 (s, 2 H, $NH_2$). $^{13}$C NMR (100 MHZ, $CDCl_3$) δ 162.7 (d, $J$ = 250 Hz), 146.4, 133.5 (d, $J$ = 8 Hz), 131.4, 129.8, 122.3, 119.0, 115.9, 115.8 (d, $J$ = 22 Hz), 109.1, 94.5, 84.4. IR ν 3474 (w), 3385 (w), 3050 (w), 1890 (w), 1616 (m), 1612 (m), 1508 (s), 1486 (s), 1411 (m), 1309 (w), 1284 (w), 1229 (s), 1155 (m), 1092 (w), 901 (w), 834 (s), 810 (s), 787 (m), 741 (w), 680 (w). HRMS (ESI) calcd for $C_{14}ClFH_{10}N^+$ $[M+H]^+$ 246.0480; found 246.0484.

**4-Chloro-2-((4-methoxyphenyl)ethynyl)aniline (2f)**

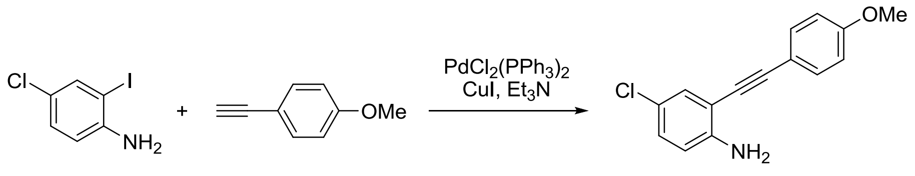


Following a slightly modified procedure [5], a solution of 4-chloro-2-iodoaniline (**4b**) (304 mg, 1.20 mmol, 1 equiv), 1-ethynyl-4-methoxybenzene (**13**) (206 mg, 1.56 mmol, 1.3 equiv), $PdCl_2(PPh_3)_2$ (84 mg, 0.12 mmol, 0.1 equiv) and CuI (23 mg, 0.12 mmol, 0.1 equiv) were heated under reflux in $Et_3N$ (12 mL) for 2 h under a nitrogen atmosphere. The resulting mixture was filtered through Celite®, washed with DCM and concentrated under vacuum. The resulting solid was purified by column chromatography (pentane/EtOAc 8/2) to afford **2f** (331 mg, 1.33 mmol, 89%) as an orange solid. Mp: 97–99 °C. Rf (pentane/EtOAc

8/2): 0.3. $^1$H NMR (400 MHz, CDCl$_3$) δ 7.46 (m, 2 H, ArH), 7.32 (d, 1 H, *J* = 2.5 Hz, ArH), 7.07 (dd, 1 H, *J* = 8.6, 2.5 Hz, ArH), 6.89 (m, 2 H, ArH), 6.64 (d, 1 H, *J* = 8.7 Hz, ArH), 4.26 (s, 2 H, NH$_2$), 3.83 (s, 3 H, CH$_3$). $^{13}$C NMR (100 MHZ, CDCl$_3$) δ 159.8, 146.2, 133.0, 131.2, 129.3, 122.2, 115.3, 114.8, 114.1, 109.7, 95.6, 83.3, 55.3. IR ν 3464 (w), 3362 (w), 3037 (w), 2932 (w), 2840 (w), 2201 (w), 1605 (s), 1567 (w), 1513 (s), 1488 (s), 1460 (w), 1410 (w), 1294 (s), 1249 (s), 1174 (m), 1152 (w), 1089 (w), 1032 (m), 901 (w), 839 (s), 818 (m), 778 (w), 737 (m). HRMS (ESI) calcd for $C_{15}ClH_{13}NO^+$ [M+H]$^+$ 258.0686; found 258.0683.

**4-Amino-3-((4-methoxyphenyl)ethynyl)benzonitrile (2g)**

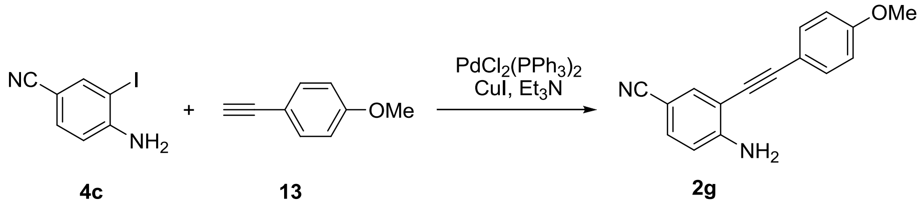


Following a slightly modified procedure [5], a solution of 4-cyano-2-iodoaniline (**4c**) (366 mg, 1.50 mmol, 1 equiv), 1-ethynyl-4-methoxybenzene (**13**) (238 mg, 1.80 mmol, 1.2 equiv), PdCl$_2$(PPh$_3$)$_2$ (102 mg, 0.150 mmol, 0.1 equiv) and CuI (28 mg, 0.15 mmol, 0.1 equiv) were heated under reflux in Et$_3$N (15 mL) for 2 h under a nitrogen atmosphere. The resulting mixture was filtered through Celite®, washed with DCM and concentrated under vacuum. The resulting solid was purified by column chromatography (pentane/EtOAc 7/3) to afford **2g** (331 mg, 1.33 mmol, 89%) as an orange solid. Mp: 138–140 °C. Rf (pentane/EtOAc 7/3): 0.3. $^1$H NMR (400 MHz, CDCl$_3$) δ 7.60 (d, 1 H, *J* = 1.5 Hz, ArH), 7.46 (d, 2 H, *J* = 8.7 Hz, ArH), 7.34 (dd, 1 H, *J* = 8.5, 1.6 Hz, ArH), 6.89 (d, 2 H, *J* = 8.7 Hz, ArH), 6.70 (d, 1 H, *J* = 8.5 Hz, ArH), 4.79 (s, 2 H, NH$_2$), 3.83 (s, 3 H, CH$_3$). $^{13}$C NMR (100 MHZ, CDCl$_3$) δ 160.0, 150.8, 136.1, 133.0, 132.9, 119.4, 114.3, 114.1, 113.8, 108.5, 100.0, 96.2, 82.0, 55.3. IR ν 3459 (m), 3354 (m), 3218 (w), 3055 (w), 2956 (w), 2838 (w), 2217 (s), 1618 (s), 1606 (s), 1559 (w), 1505 (s), 1467 (w), 1423 (w), 1337 (w), 1288 (m), 1248 (s), 1174 (m), 1158 (w), 1109 (w), 1026 (m), 907 (w), 832 (s), 787 (w), 736 (w). HRMS (ESI) calcd for $C_{16}H_{13}N_2O^+$ [M+H]$^+$ 249.1022; found 249.1014.

**2-(Hex-1-yn-1-yl)aniline (2h)**

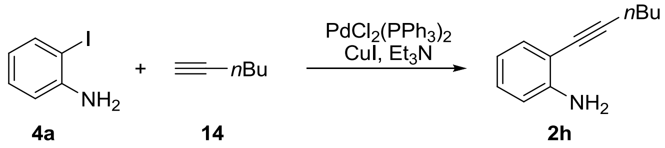


Following a slightly modified procedure [5], a solution of 2-iodoaniline (**4a**) (438 mg, 2.00 mmol, 1 equiv), 1-hexyne (**14**) (277 µL, 2.4 mmol, 1.2 equiv), PdCl$_2$(PPh$_3$)$_2$ (135 mg, 0.200 mmol, 0.1 equiv) and CuI (38 mg, 0.20 mmol, 0.1 equiv) were heated under reflux in

$Et_3N$ (20 mL) for 2 h under a nitrogen atmosphere. The resulting mixture was filtered through Celite®, washed with DCM and concentrated under vacuum. The resulting solid was purified by column chromatography (pentane/EtOAc 98/2) to afford **2h** (293 mg, 1.69 mmol, 85%) as a yellow liquid. Rf (pentane/EtOAc 98/2): 0.15. $^1H$ NMR (400 MHz, $CDCl_3$) δ 7.26 (dd, 1 H, *J* = 7.5, 1.4 Hz, ArH), 7.09 (m, 1 H, ArH), 6.68 (m, 2 H, ArH), 4.17 (br s, 2 H, $NH_2$), 2.49 (t, 2 H, *J* = 7.0 Hz, $CH_2$), 1.62 (m, 2 H, $CH_2$), 1.51 (m, 2 H, $CH_2$), 0.97 (t, 3 H, *J* = 7.3 Hz, $CH_3$). $^{13}C$ NMR (100 MHZ, $CDCl_3$) δ 147.7, 132.1, 128.8, 117.9, 114.2, 109.0, 95.8, 77.0, 31.1, 22.1, 19.4, 13.7. Consistent with reported values [6].

**2-((Trimethylsilyl)ethynyl)aniline (2i)**

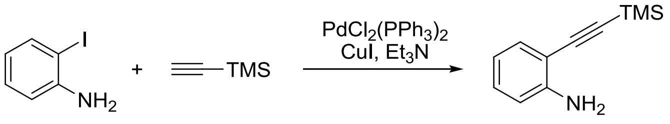


Following a slightly modified procedure [5], a solution of 2-iodoaniline (**4a**) (438 mg, 2.00 mmol, 1 equiv), trimethylsilyl acetylene (**15**) (342 µL, 2.40 mmol, 1.2 equiv), $PdCl_2(PPh_3)_2$ (135 mg, 0.200 mmol, 0.1 equiv) and CuI (38 mg, 0.20 mmol, 0.1 equiv) were heated under reflux in $Et_3N$ (20 mL) for 2 h under a nitrogen atmosphere. The resulting mixture was filtered through Celite®, washed with DCM and concentrated under vacuum. The resulting solid was purified by column chromatography (pentane/EtOAc 98/2) to afford **2i** (242 mg, 1.28 mmol, 64%) as a colorless liquid. Rf (pentane/EtOAc 98/2): 0.25.$^1H$ NMR (400 MHz, $CDCl_3$) δ 7.31 (dd, *J* = 7.5, 1.3 Hz, 1 H, ArH), 7.12 (dt, *J* = 7.8, 1.6 Hz, ArH), 6.67 (m, 2 H, ArH), 4.24 (br s, 2 H, $NH_2$), 0.29 (s, 9 H, TMS).$^{13}C$ NMR (100 MHz, $CDCl_3$) δ 148.2, 132.2, 129.8, 117.7, 114.1, 107.7, 101.8, 99.7, 0.1. Consistent with reported values [7].

**2-(Ethynyl)aniline (2j)**

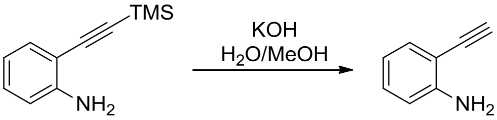


KOH (1M in $H_2O$, 700 µL, 0.700 mmol, 1.1 equiv) was added to a stirred solution of **2i** (121mg, 0.640 mmol, 1 equiv) in MeOH (2 mL). After 1 h, the reaction was diluted in DCM (20 mL) and water (20 mL). The layers were separated and the aqueous layer extracted with DCM (20 mL). The organic layers were combined, washed with brine (20 mL), dried over $MgSO_4$, filtered and concentrated under vacuum to afford **2j** (69 mg, 0.59 mmol, 92%) as a yellow oil. $^1H$ NMR (400 MHz, $CDCl_3$) δ 7.35 (dd, 1 H, *J* = 8.0, 1.6 Hz, ArH), 7.16 (td, *J* = 7.8, 1.5 Hz, 1 H, ArH), 6.70 (m, 2 H, ArH), 4.26 (br s, 2 H, $NH_2$), 3.40 (s, 1 H, CH).

$^{13}$C NMR (100 MHZ, $CDCl_3$) δ 148.5, 132.5, 130.1, 117.7, 114.2, 106.5, 82.4, 80.6. Consistent with reported values [8].

## Sequential annulations/direct alkynylation

### 2-Phenyl-3-((triisopropylsilyl)ethynyl)-1*H*-indole (3a)

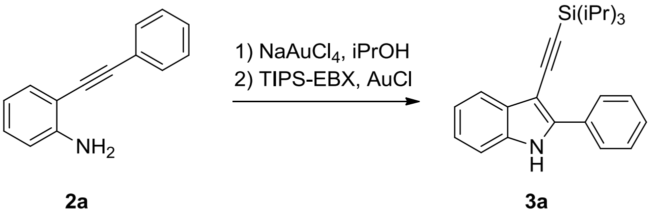


$NaAuCl_4$ (3.2 mg, 0.0081 mmol, 0.02 equiv) was added to a stirred solution of **2a** (78 mg, 0.40 mmol, 1 equiv) in iPrOH (3 mL) under an ambient atmosphere. The reaction was stirred at RT for 3 h. TIPS-EBX (**1**) (206 mg, 0.480 mmol, 1.2 equiv) and then AuCl (3.7 mg, 0.016 mmol, 0.04 equiv) were added. The reaction was stirred for 30 h and then concentrated under vacuum. $Et_2O$ (20 mL) was added, the organic layer washed twice with 0.1 M NaOH (20 mL). The aqueous layers were combined and extracted with $Et_2O$ (20 mL). The organic layers were combined, washed successively with saturated $NaHCO_3$ (20 mL) and brine (20 mL), dried with $MgSO_4$ and concentrated under reduced pressure. Purification by flash chromatography (pentane/$Et_2O$ 8/2) afforded **3a** (1. run: 145 mg, 0.388 mmol, 97%, 2. run: 142 mg, 0.380 mmol, 95%) as a brown amorphous solid. Rf (pentane/$Et_2O$ 8/2): 0.4. $^{1}$H NMR ($CDCl_3$, 400 MHz) δ 8.32 (br s, 1 H; NH), 8.11 (m, 2 H; ArH), 7.80 (m, 1 H; ArH), 7.49 (m, 2 H; ArH), 7.40 (m, 2 H; ArH), 7.27 (m, 2H; ArH), 1.24 (m, 21 H; TIPS). $^{13}$C NMR ($CDCl_3$, 100 MHz) δ 139.7, 135.1, 131.3, 130.8, 128.7, 128.3, 126.4, 123.4, 120.9, 120.1, 110.9, 101.2, 96.5, 95.1, 18.8, 11.5. Consistent with reported values [9].

### 2-(*p*-Tolyl)-3-((triisopropylsilyl)ethynyl)-1*H*-indole (3b)

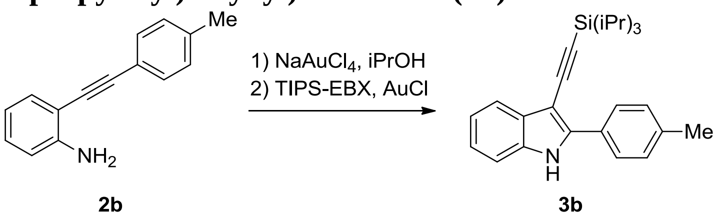


$NaAuCl_4$ (3.2 mg, 0.0081 mmol, 0.02 equiv) was added to a stirred solution of **2b** (83 mg, 0.40 mmol, 1 equiv) in iPrOH (3 mL) under an ambient atmosphere. The reaction was stirred at RT for 3 h. TIPS-EBX (**1**) (206 mg, 0.480 mmol, 1.2 equiv) and then AuCl (3.7 mg, 0.016 mmol, 0.04 equiv) were added. The reaction was stirred for 24 h and then concentrated under vacuum. $Et_2O$ (20 mL) was added, the organic layer washed twice with 0.1 M NaOH (20

mL). The aqueous layers were combined and extracted with $Et_2O$ (20 mL). The organic layers were combined, washed successively with saturated $NaHCO_3$ (20 mL) and brine (20 mL), dried with $MgSO_4$ and concentrated under reduced pressure. Purification by flash chromatography (pentane/$Et_2O$ 9/1) afforded **3b** (115 mg, 0.296 mmol, 74%) as an orange amorphous solid. Rf (pentane/$Et_2O$ 9/1): 0.2. $^1$H NMR (400 MHz, $CDCl_3$) δ 8.26 (s, 1 H, NH), 8.00 (d, 2 H, *J* = 8.2 Hz, ArH), 7.75 (d, 1 H, *J* = 6.9 Hz, ArH), 7.36 (m, 1 H, ArH), 7.25 (m, 4 H, ArH), 2.42 (s, 3 H, $CH_3$), 1.21 (m, 21 H, TIPS). $^{13}$C NMR (100 MHZ, $CDCl_3$) δ 139.8, 138.4, 135.0, 130.9, 129.4, 128.6, 126.2, 123.3, 120.9, 120.0, 110.8, 101.4, 96.0, 95.0, 21.4, 18.8, 11.5. IR ν 3418 (m), 3063 (w), 2942 (s), 2863 (s), 2141 (s), 1676 (w), 1617 (w), 1504 (w), 1458 (s), 1382 (w), 1327 (m), 1305 (w), 1235 (m), 1175 (w), 1115 (w), 1060 (w), 1010 (w), 997 (m), 910 (m), 883 (m), 821 (m), 780 (m), 743 (s), 677 (s). HRMS (ESI) calcd for $C_{26}H_{34}NSi^+$ $[M+H]^+$ 388.2455; found 388.2459

**2-(4-Fluorophenyl)-3-((triisopropylsilyl)ethynyl)-1*H*-indole (3c)**

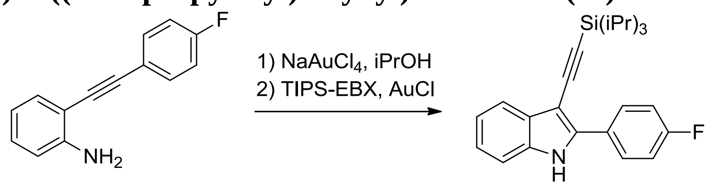


**2c** **3c**

$NaAuCl_4$ (3.2 mg, 0.0081 mmol, 0.02 equiv) was added to a stirred solution of **2c** (84 mg, 0.40 mmol, 1 equiv) in iPrOH (3 mL) under an ambient atmosphere. The reaction was stirred at RT for 3 h. TIPS-EBX (**1**) (206 mg, 0.480 mmol, 1.2 equiv) and then AuCl (3.7 mg, 0.016 mmol, 0.04 equiv) were added. The reaction was stirred for 30 h and then concentrated under vacuum. $Et_2O$ (20 mL) was added, the organic layer washed twice with 0.1 M NaOH (20 mL). The aqueous layers were combined and extracted with $Et_2O$ (20 mL). The organic layers were combined, washed successively with saturated $NaHCO_3$ (20 mL) and brine (20 mL), dried with $MgSO_4$ and concentrated under reduced pressure. Purification by flash chromatography (pentane/$Et_2O$ 8/2) afforded **3c** (124 mg, 0.317 mmol, 79%) as brown amorphous solid. Rf (pentane/$Et_2O$ 8/2): 0.4. $^1$H NMR (400 MHz, $CDCl_3$) δ 8.08 (s, 1 H, NH), 7.93 (dd, 2 H, *J* = 8.9, 5.3 Hz, ArH), 7.65 (m, 1 H, ArH), 7.25 (m, 1 H, ArH), 7.14 (m, 2 H, ArH), 7.04 (t, 2 H, *J* = 8.6 Hz, ArH), 1.10 (m, 21 H, TIPS). $^{13}$C NMR (100 MHZ, $CDCl_3$) δ 162.7 (d, *J* = 249 Hz), 138.8, 135.1, 130.7, 128.4 (d, *J* = 8 Hz), 127.7 (d, *J* = 3 Hz), 123.6, 121.1, 120.1, 115.9 (d, *J* = 22 Hz), 110.9, 101.0, 96.5, 95.2, 18.8, 11.5. IR ν 3422 (w), 2864 (m), 1890 (w), 1546 (w), 1502 (m), 1457 (m), 1440 (m), 1367 (w), 1327 (m), 1235 (s), 1162 (m), 1153 (w), 1104 (w), 1059 (w), 996 (m), 908 (m), 883 (m), 836 (s), 781 (m), 781 (m), 742 (s), 679 (s). HRMS (ESI) calcd for $C_{25}FH_{31}NSi^+$ $[M+H]^+$ 392.2204; found 392.2195

**2-(4-Methoxyphenyl)-3-((triisopropylsilyl)ethynyl)-1*H*-indole (3d)**

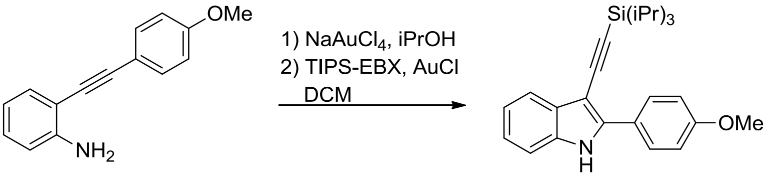


$NaAuCl_4$ (3.2 mg, 0.0081 mmol, 0.02 equiv) was added to a stirred solution of **2d** (89 mg, 0.40 mmol, 1 equiv) in iPrOH (3 mL) under an ambient atmosphere. The reaction was stirred at RT for 3 h. DCM (1.5 mL), TIPS-EBX (**1**) (206 mg, 0.480 mmol, 1.2 equiv) and then AuCl (3.7 mg, 0.016 mmol, 0.04 equiv) were added. The reaction was stirred for 18 h and then concentrated under vacuum. $Et_2O$ (20 mL) was added, the organic layer washed twice with 0.1 M NaOH (20 mL). The aqueous layers were combined and extracted with $Et_2O$ (20 mL). The organic layers were combined, washed successively with saturated $NaHCO_3$ (20 mL) and brine (20 mL), dried with $MgSO_4$ and concentrated under reduced pressure. Purification by flash chromatography (pentane/$Et_2O$ 8/2) afforded **3d** (128 mg, 0.317 mmol, 79%) as a brown oil. Rf (pentane/$Et_2O$ 8/2): 0.4. $^1$H NMR (400 MHz, $CDCl_3$) δ 8.22 (s, 1 H, NH), 8.04 (m, 2 H, ArH), 7.75 (m, 1 H, ArH), 7.36 (m, 1 H, ArH), 7.24 (m, 2 H, ArH), 7.00 (m, 2 H, ArH), 3.90 (s, 3 H. $CH_3$), 1.22 (m, 21H, TIPS). $^{13}$C NMR (100 MHZ, $CDCl_3$) δ 159.7, 139.9, 135.0, 130.9, 129.6, 127.8, 124.1, 123.1, 119.9, 114.1, 110.7, 101.5, 95.4, 94.7, 55.4, 18.8, 11.5. IR ν 3415 (w), 3063 (w), 2863 (m), 2140 (m), 1612 (m), 1578 (w), 1545 (w), 1504 (s), 1458 (s), 1439 (m), 1367 (w), 1328 (w), 1308 (m), 1285 (m), 1255 (s), 1184 (m), 1116 (w), 1062 (w), 1032 (m), 1019 (w), 911 (w), 883 (m), 834 (m), 790 (m), 744 (s), 678 (m), 659 (m). HRMS (ESI) calcd for $C_{26}H_{34}NOSi^+$ $[M+H]^+$ 404.2404; found 404.2423.

**5-Chloro-2-(4-fluorophenyl)-3-((triisopropylsilyl)ethynyl)-1*H*-indole (3e)**

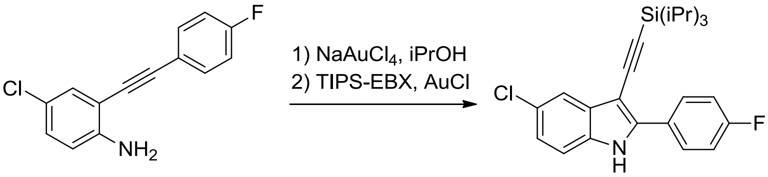


$NaAuCl_4$ (3.2 mg, 0.0081 mmol, 0.02 equiv) was added to a stirred solution of **2e** (98 mg, 0.40 mmol, 1 equiv) in iPrOH (3 mL) under an ambient atmosphere. The reaction was stirred at RT for 3 h. TIPS-EBX (**1**) (206 mg, 0.480 mmol, 1.2 equiv) and then AuCl (3.7 mg, 0.016 mmol, 0.04 equiv) were added. The reaction was stirred for 30 h and then concentrated under vacuum. $Et_2O$ (20 mL) was added, the organic layer washed twice with 0.1 M NaOH (20 mL). The aqueous layers were combined and extracted with $Et_2O$ (20 mL). The organic layers were combined, washed successively with saturated $NaHCO_3$ (20 mL) and brine (20 mL),

dried with $MgSO_4$ and concentrated under reduced pressure. Purification by flash chromatography (pentane/$Et_2O$ 9/1 to 7/3) afforded **3e** (124 mg, 0.291 mmol, 73%) as a grey solid. Mp: 99–100 °C. Rf (pentane/$Et_2O$ 9/1): 0.2. $^1H$ NMR (400 MHz, $CDCl_3$) δ 8.25 (s, 1 H, NH), 8.03 (m, 2 H, ArH), 7.68 (d, 1 H, *J* = 1.5 Hz, ArH), 7.27 (m, 1 H, ArH), 7.18 (m, 3 H, ArH), 1.21 (m, 21 H, TIPS). $^{13}C$ NMR (100 MHZ, $CDCl_3$) δ 162.8 (d, *J* = 249 Hz), 140.1, 133.4, 131.7, 128.4 (d, *J* = 8 Hz), 127.2 (d, *J* = 3 Hz), 126.9, 123.9, 119.6, 115.9 (d, *J* = 22 Hz), 112.0, 100.1, 96.2, 95.8, 18.8, 11.4. IR ν 2926 (s), 2848 (s), 2106 (w), 1587 (w), 1530 (w), 1486 (m), 1433 (s), 1344 (w), 1294 (w), 1260 (m), 1200 (w), 1141 (w), 1089 (w), 1052 (w), 961 (w), 893 (s), 863 (m), 827 (m), 774 (w), 759 (m), 707 (s). HRMS (ESI) calcd for $C_{25}ClFH_{30}NSi^+$ $[M+H]^+$ 426.1815; found 426.1824.

**5-Chloro-2-(4-methoxyphenyl)-3-((triisopropylsilyl)ethynyl)-1*H*-indole (3f)**

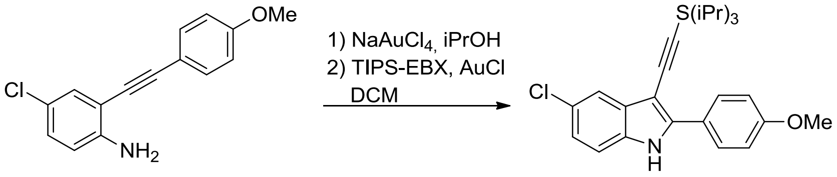

**2f** **3f**

$NaAuCl_4$ (3.2 mg, 0.0081 mmol, 0.02 equiv) was added to a stirred solution of **2f** (103 mg, 0.400 mmol, 1 equiv) in iPrOH (3 mL) under an ambient atmosphere. The reaction was stirred at RT for 3 h. DCM (1.5 mL), TIPS-EBX (**1**) (206 mg, 0.480 mmol, 1.2 equiv) and then AuCl (7.4 mg, 0.032 mmol, 0.08 equiv) were added. The reaction was stirred for 30 h and then concentrated under vacuum. $Et_2O$ (20 mL) was added, the organic layer washed twice with 0.1 M NaOH (20 mL). The aqueous layers were combined and extracted with $Et_2O$ (20 mL). The organic layers were combined, washed successively with saturated $NaHCO_3$ (20 mL) and brine (20 mL), dried with $MgSO_4$ and concentrated under reduced pressure. Purification by flash chromatography (pentane/$Et_2O$ 8/2) afforded **3f** (99 mg, 0.23 mmol, 56%, 90% pure) as a grey solid. Analytically pure product was obtained by preparative TLC (pentane/$Et_2O$ 7/3). Rf (pentane/$Et_2O$ 7/3): 0.2. Mp: 180–182 °C. $^1H$ NMR (400 MHz, $CDCl_3$) δ 8.24 (s, 1 H, NH), 8.01 (m, 2 H, ArH), 7.65 (d, 1 H, *J* = 2.0 Hz, ArH), 7.27 (m, 1 H, ArH), 7.18 (dd, 1 H, *J* = 8.5, 2.0 Hz, ArH), 6.99 (m, 2 H, ArH), 3.88 (s, 3 H, $CH_3$), 1.19 (m, 21 H, TIPS). $^{13}C$ NMR (100 MHZ, $CDCl_3$) δ 160.0, 141.2, 133.3, 132.0, 127.9, 126.7, 123.6, 123.4, 119.4, 114.2, 111.7, 100.6, 98.4, 95.3, 55.4, 18.8, 11.5. IR ν 3427 (w), 2865 (w), 2141 (w), 1545 (w), 1503 (m), 1467 (s), 1311 (w), 1288 (m), 1256 (s), 1183 (m), 1073 (w), 1033 (m), 997 (w), 910 (m), 883 (w), 837 (m), 803 (s), 735 (m), 697 (m), 667 (m). HRMS (ESI) calcd for $C_{26}ClH_{33}NOSi^+$ $[M+H]^+$ 438.2014; found 438.2018.

**2-(4-Methoxyphenyl)-3-((triisopropylsilyl)ethynyl)-1*H*-indole-5-carbonitrile (3g)**

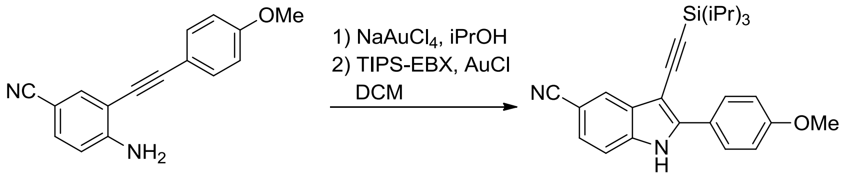

2g 3g

$NaAuCl_4$ (6.4 mg, 0.016 mmol, 0.04 equiv) was added to a stirred solution of **2g** (99 mg, 0.40 mmol, 1 equiv) in iPrOH (3 mL) under an ambient atmosphere. The reaction was stirred at 80°C for 3 h. The reaction was cooled to RT and DCM (1.5 mL), TIPS-EBX (**1**) (206 mg, 0.480 mmol, 1.2 equiv) and then AuCl (7.4 mg, 0.032 mmol, 0.08 equiv) were added. The reaction was stirred for 18 h and then TIPS-EBX (206 mg, 0.480 mmol, 1.2 equiv) added. After 12 h the reaction was concentrated under vacuum. $Et_2O$ (20 mL) was added, the organic layer washed twice with 0.1 M NaOH (20 mL). The aqueous layers were combined and extracted with $Et_2O$ (20 mL). The organic layers were combined, washed successively with saturated $NaHCO_3$ (20 mL) and brine (20 mL), dried with $MgSO_4$ and concentrated under reduced pressure. Purification by flash chromatography (pentane/$Et_2O$ 5/5 to 3/7) afforded **3g** (98 mg, 0.23 mmol, 54%, 95% pure) as a grey solid. A second batch of product **3g** (48 mg, 0.11 mmol, 25%, 90% pure) was also obtained. Analytically pure product was obtained by recrystallization from hexanes/EtOAc. Combined yield: 79%. Rf (pentane/$Et_2O$ 5/5): 0.2. Mp: 158–159 °C. $^1H$ NMR (400 MHz, $CDCl_3$) δ 8.97 (s, 1 H, NH), 8.04 (m, 2 H, ArH), 8.00 (m, 1 H, ArH), 7.42 (m, 2 H, ArH), 6.97 (m, 2 H, ArH), 3.85 (m, 3 H, $CH_3$), 1.20 (m, 21 H, TIPS). $^{13}C$ NMR (100 MHZ, $CDCl_3$) δ 160.3, 142.0, 136.7, 130.7, 128.1, 126.0, 125.0, 123.0, 120.7, 114.3, 111.7, 103.6, 99.8, 96.4, 95.7, 55.4, 18.8, 11.4. IR ν 3310 (m), 2941 (m), 2864 (m), 2223 (m), 2142 (m), 1707 (w), 1615 (m), 1586 (w), 1546 (w), 1504 (s), 1473 (s), 1382 (w), 1293 (m), 1255 (s), 1184 (s), 1133 (w), 1034 (m), 1025 (w), 909 (m), 884 (m), 833 (m), 805 (m), 739 (s), 672 (m), 661 (m). HRMS (ESI) calcd for $C_{27}H_{33}N_2OSi^+$ $[M+H]^+$ 429.2357; found 429.2369.

**2-Butyl-3-((triisopropylsilyl)ethynyl)-1*H*-indole (3h)**

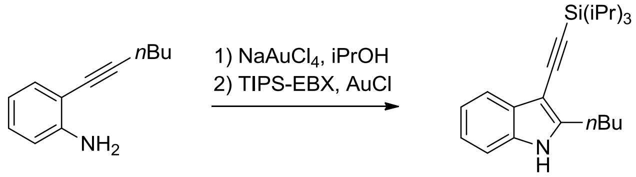

2h 3h

$NaAuCl_4$ (3.2 mg, 0.0081 mmol, 0.02 equiv) was added to a stirred solution of **2h** (69 mg, 0.40 mmol, 1 equiv) in iPrOH (3 mL) under an ambient atmosphere. The reaction was stirred at RT for 3 h. TIPS-EBX (**1**) (206 mg, 0.480 mmol, 1.2 equiv) and then AuCl (3.7 mg, 0.016

mmol, 0.04 equiv) were added. The reaction was stirred for 4 h and the concentrated under vacuum. $Et_2O$ (20 mL) was added, the organic layer washed twice with 0.1 M NaOH (20 mL). The aqueous layers were combined and extracted with $Et_2O$ (20 mL). The organic layers were combined, washed successively with saturated $NaHCO_3$ (20 mL) and brine (20 mL), dried with $MgSO_4$ and concentrated under reduced pressure. Purification by flash chromatography (pentane/$Et_2O$ 9/1) afforded **3h** (120 mg, 0.339 mmol, 85%) as yellow oil. Rf (pentane/$Et_2O$ 9/1): 0.4. $^1$H NMR (400 MHz, $CDCl_3$) δ 7.88 (s, 1 H, NH), 7.65 (m, 1 H, ArH), 7.27 (m, 1H, ArH), 7.16 (m, 2 H, ArH), 2.90 (t, 2 H, $J$ = 7.5 Hz, $CH_2$), 1.74 (m, 2 H, $CH_2$), 1.41 (m, 2 H, $CH_2$), 1.18 (m, 21 H, TIPS), 0.95 (t, 3 H, $J$ = 7.3 Hz, $CH_3$). $^{13}$C NMR (100 MHZ, $CDCl_3$) δ 144.7, 134.4, 129.5, 122.1, 120.5, 119.4, 110.5, 100.6, 96.7, 93.1, 31.2, 27.1, 22.3, 18.8, 13.8, 11.4. IR ν 3401 (m), 2958 (s), 2942 (s), 2865 (s), 2146 (s), 1617 (w), 1548 (w), 1460 (s), 1382 (w), 1330 (w), 1242 (m), 1158 (w), 1077 (w), 1000 (w), 997 (w), 920 (w), 883 (m), 778 (m), 743 (s), 677 (s), 631 (s). HRMS (ESI) calcd for $C_{23}H_{36}NSi^+$ $[M+H]^+$ 354.2612; found 354.2606.

## References


1. Kraszkiewicz, L.; Skulski, L. *Arkivoc* **2003,** *6,* 120–125.
2. Helal, C J.; Magriotis, P. A; Corey, E. J. *J. Am. Chem. Soc.* **1996,** *118,* 10938–10939. doi:10.1021/ja962849e
   Zhdankin, V. V.; Kuehl, C. J.; Krasutsky, A. P.; Bolz, J. T.; Simonsen, A. J. *J. Org. Chem.* **1996,** *61,* 6547–6551. doi:10.1021/jo960927a
3. Yin, Y.; Ma, W. Y.; Chai, Z.; Zhao, G. *J. Org. Chem.* **2007,** *72,* 5731–5736. doi:10.1021/jo070681h
4. Swamy, N. K.; Yazici, A.; Pyne, S. G. *J. Org. Chem.* **2010,** *75,* 3412–3419. doi:10.1021/jo1005119
5. Yanada, R.; Hashimoto, K.; Tokizane, R.; Miwa, Y.; Minami, H.; Yanada, K.; Ishikura, M.; Takemoto, Y. *J. Org. Chem.* **2008,** *73,* 5135–5138. doi:10.1021/jo800474c
6. Yamane, Y.; Liu, X. H.; Hamasaki, A.; Ishida, T.; Haruta, M.; Yokoyama, T.; Tokunaga, M. *Org. Lett.* **2009,** *11,* 5162–5165. doi:10.1021/ol902061j
7. Sakai, N.; Annaka, K.; Konakahara, T. *J. Org. Chem. 2006, 71,* 3653–3655. doi:10.1021/jo060245f

# Spectra of new compounds

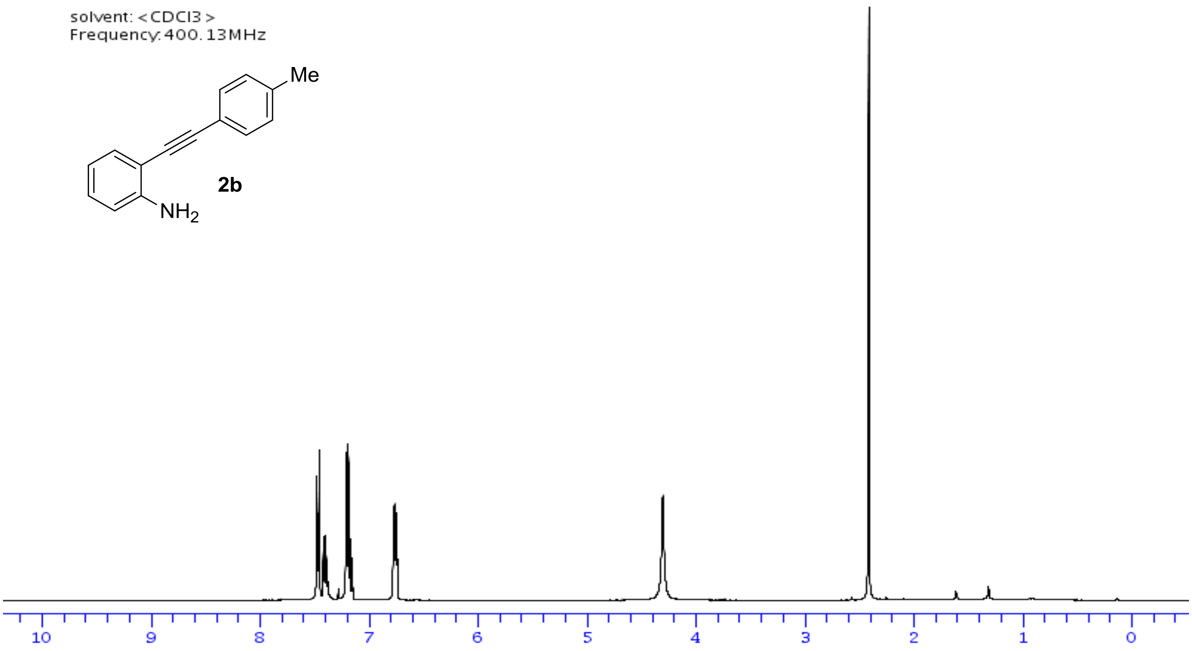


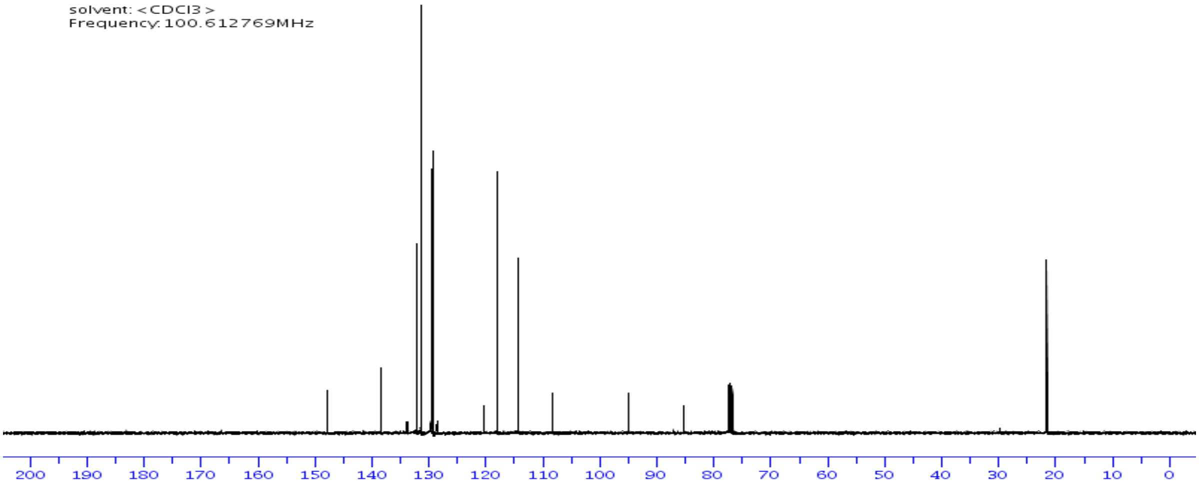


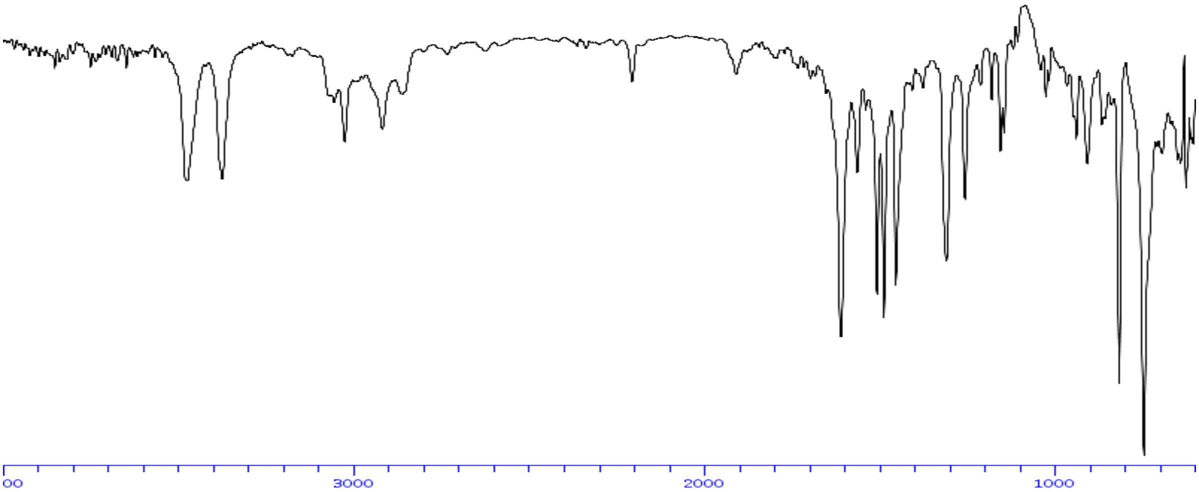

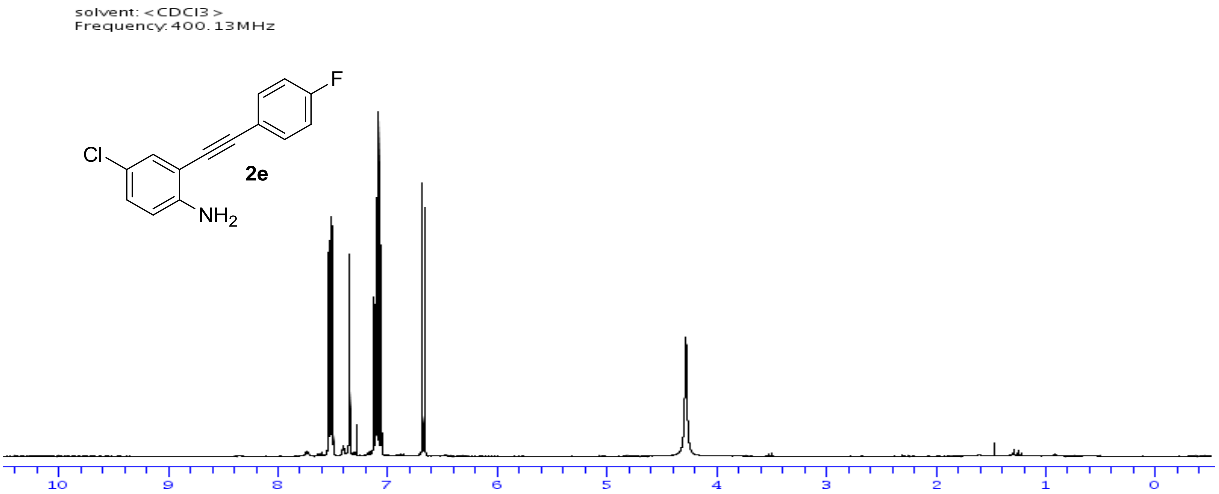
solvent: <CDCl3>
Frequency: 400.13MHz
F
Cl
2e
NH2

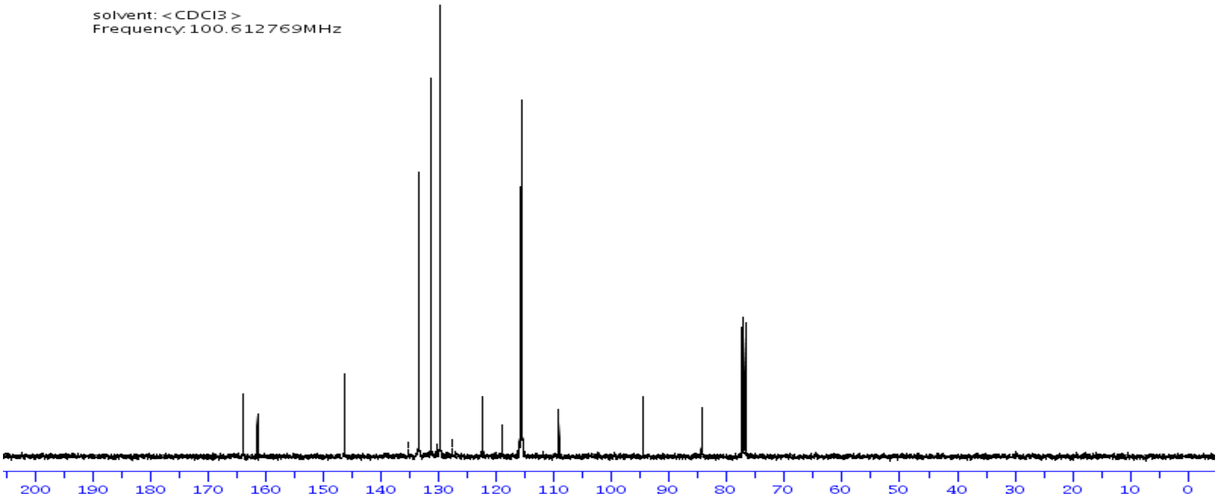
solvent: <CDCl3>
Frequency: 100.612769MHz

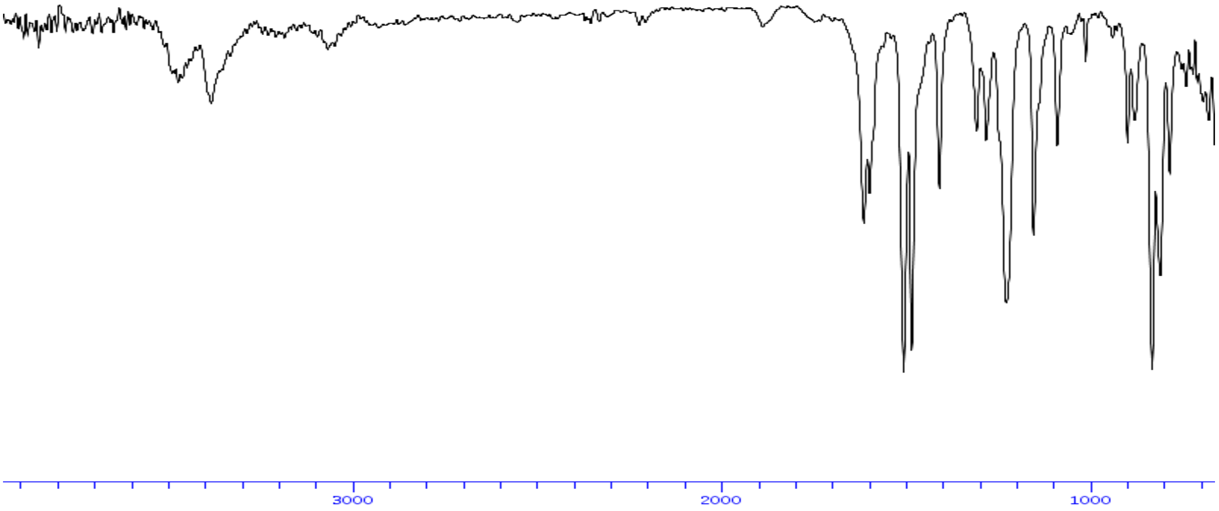

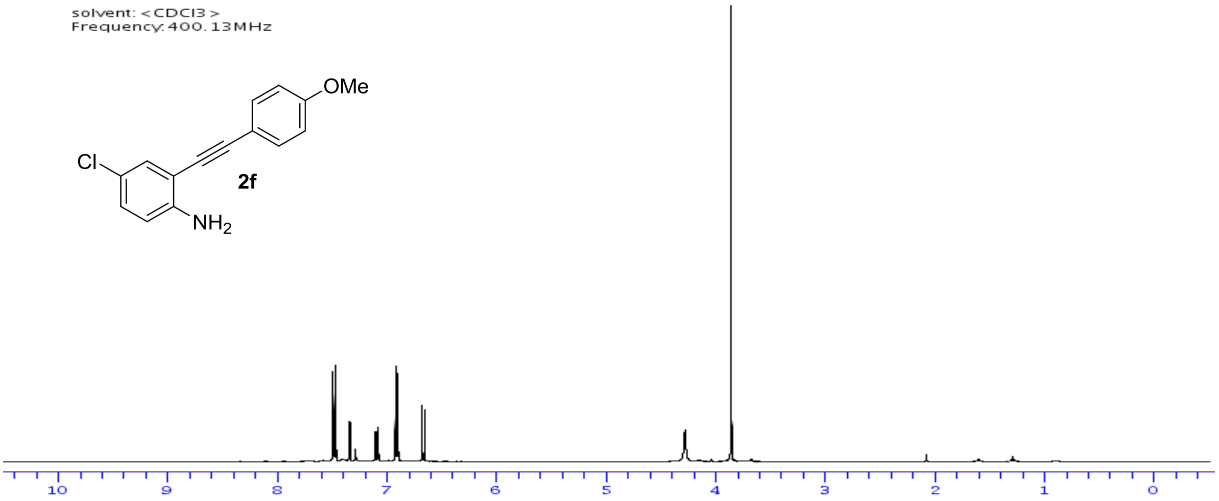
solvent: <CDCl3>
Frequency: 400.13MHz
OMe
Cl
2f
NH2
10 9 8 7 6 5 4 3 2 1 0

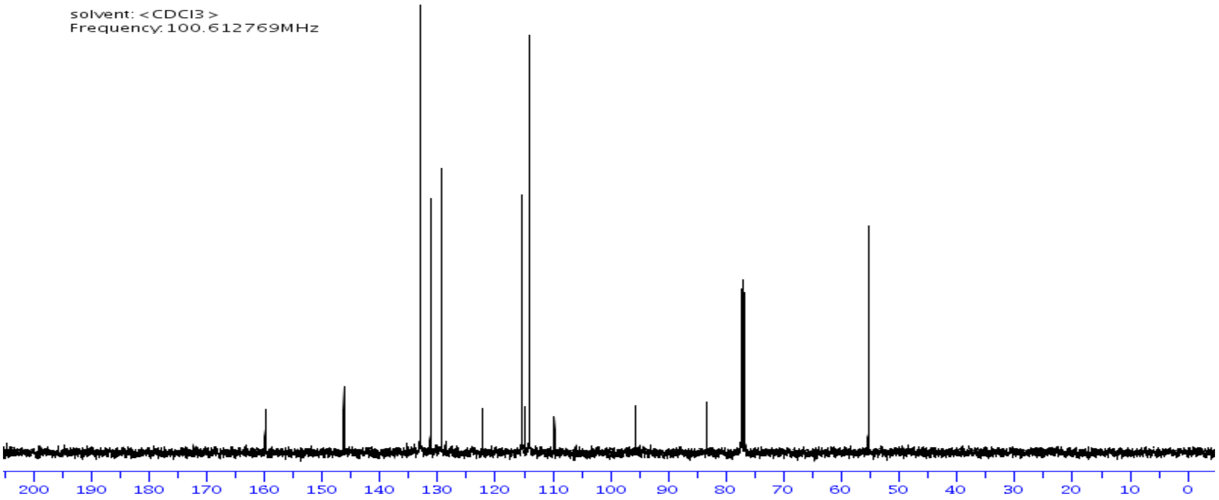
solvent: <CDCl3>
Frequency: 100.612769MHz
200 190 180 170 160 150 140 130 120 110 100 90 80 70 60 50 40 30 20 10 0

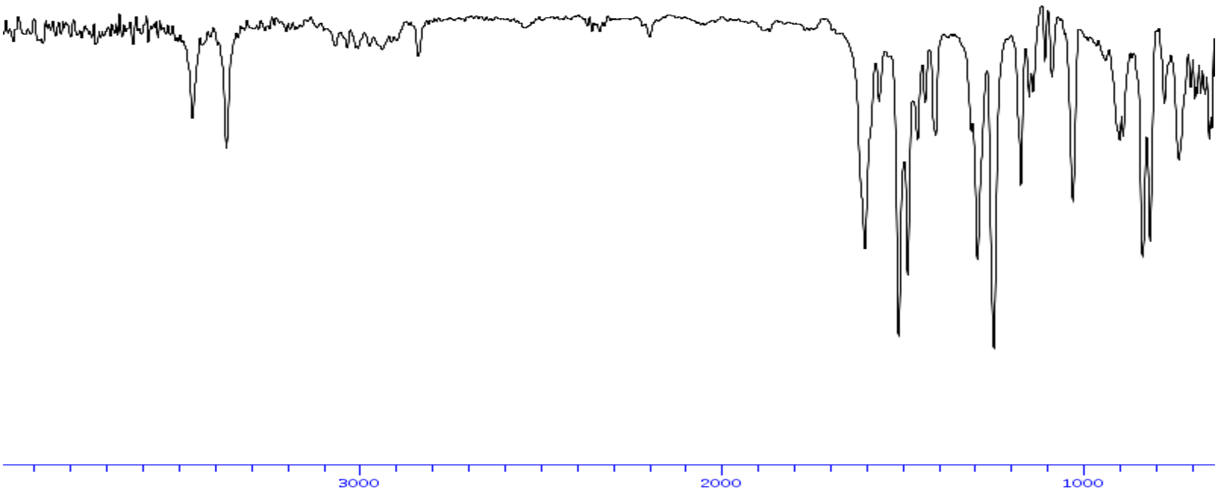
3000 2000 1000

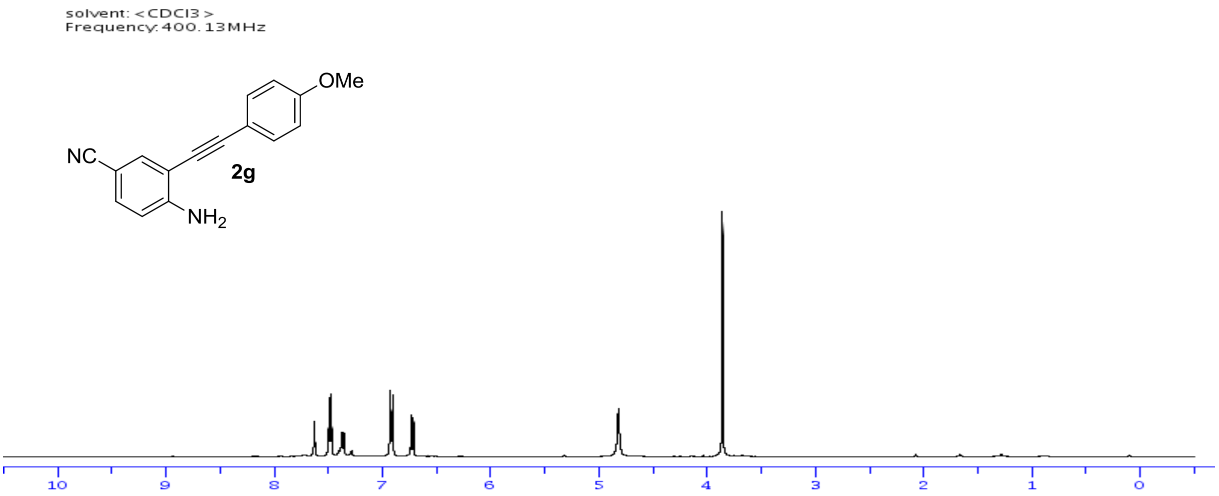
solvent: <CDCl3>
Frequency: 400.13MHz
OMe
NC
2g
NH2
10 9 8 7 6 5 4 3 2 1 0

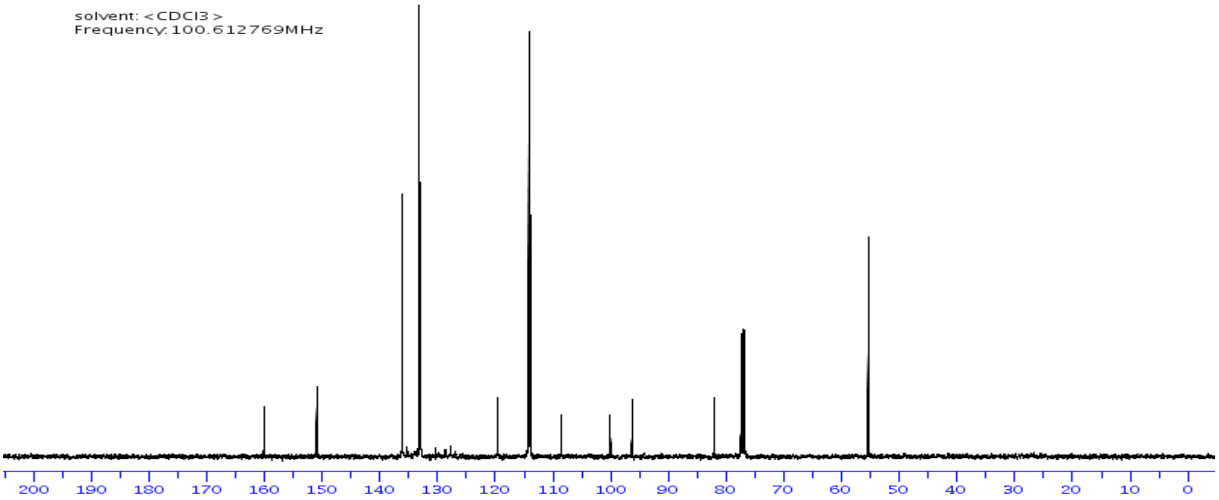
solvent: <CDCl3>
Frequency: 100.612769MHz
200 190 180 170 160 150 140 130 120 110 100 90 80 70 60 50 40 30 20 10 0

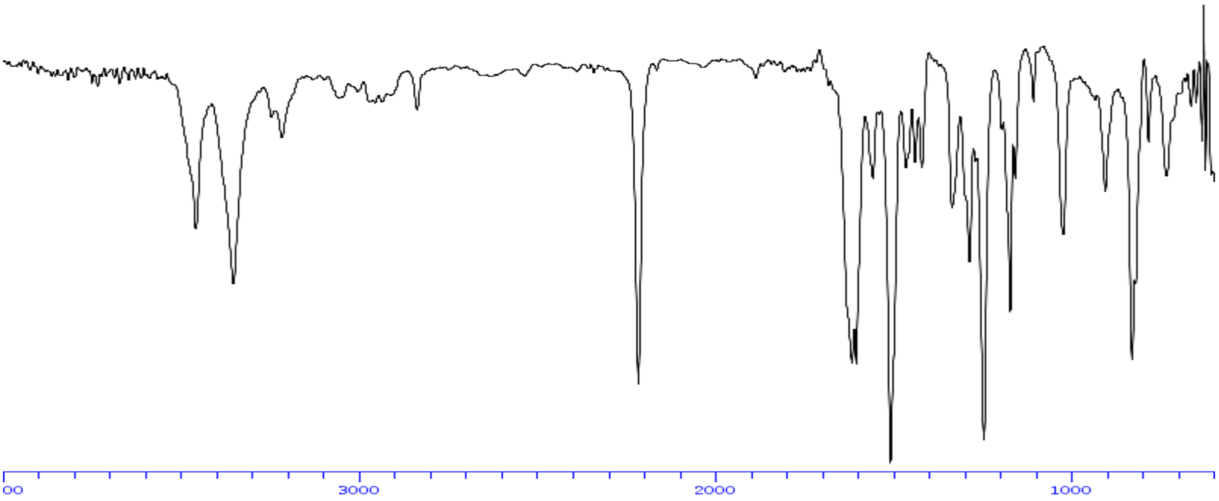
00 3000 2000 1000

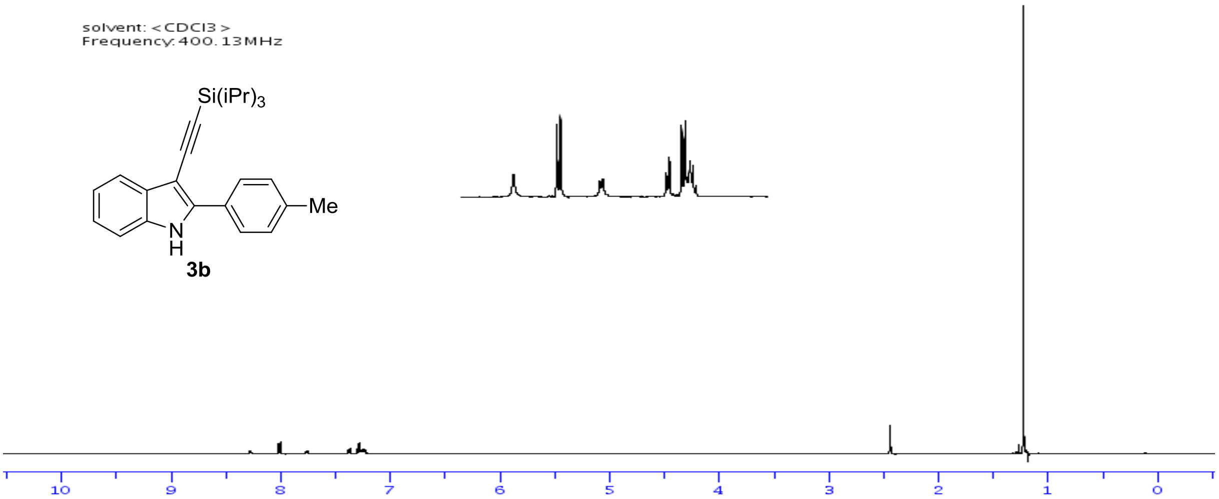
solvent: <CDCl3>
Frequency: 400.13MHz
Si(iPr)3
Me
N
H
3b
10 9 8 7 6 5 4 3 2 1 0

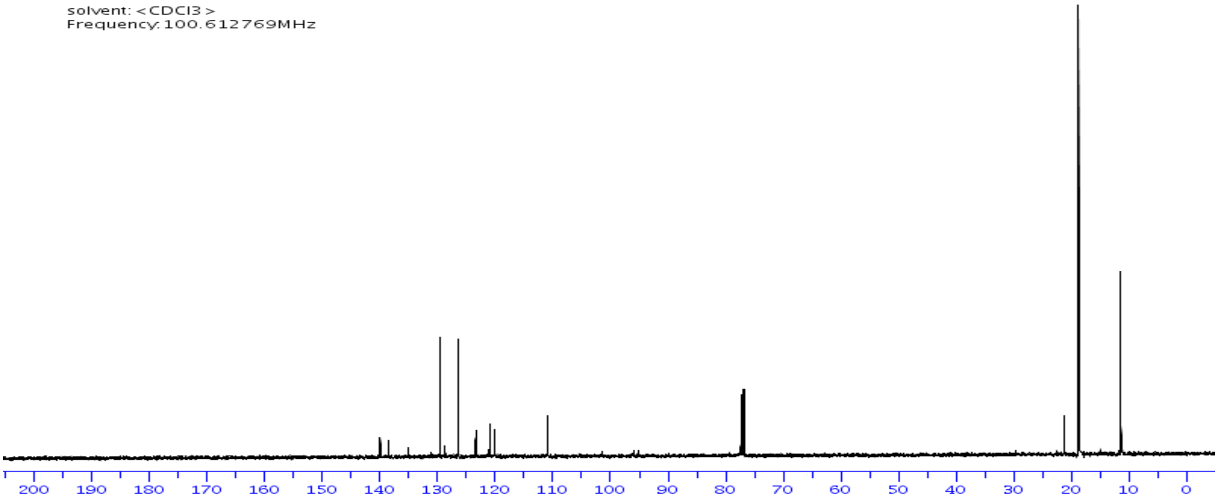
solvent: <CDCl3>
Frequency: 100.612769MHz
200 190 180 170 160 150 140 130 120 110 100 90 80 70 60 50 40 30 20 10 0

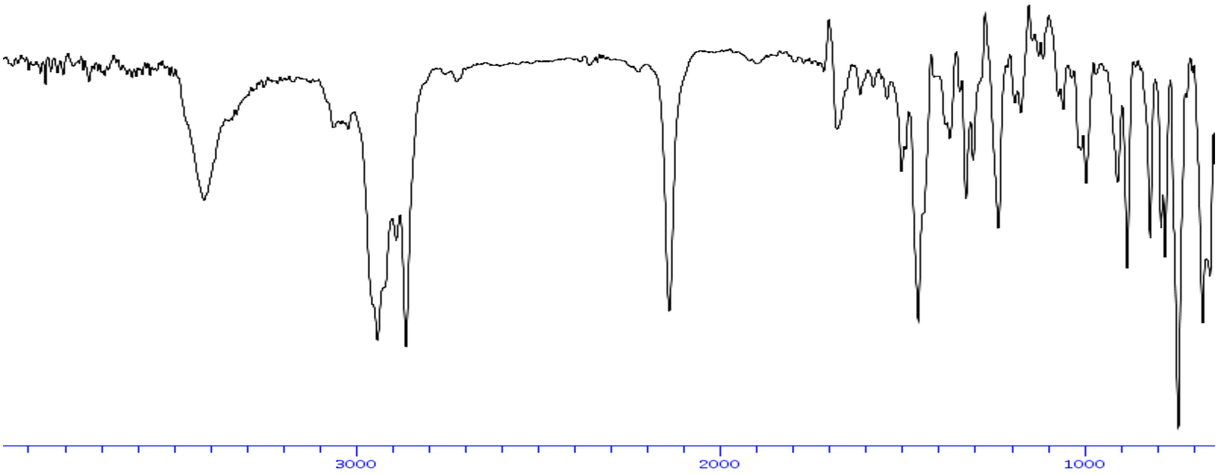
3000
2000
1000

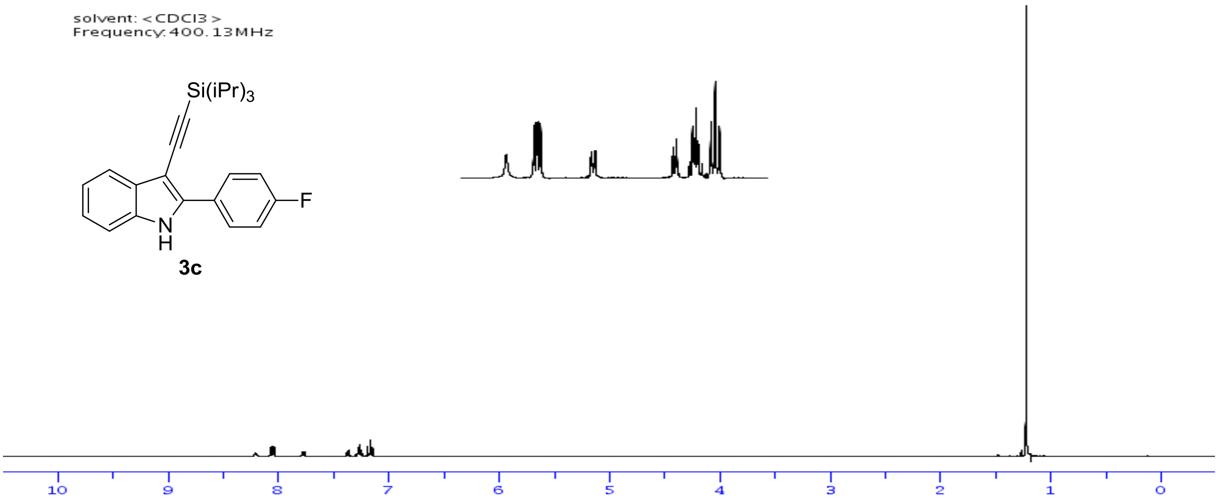
solvent: <CDCl3>
Frequency: 400.13MHz
Si(iPr)3
F
N
H
3c
10 9 8 7 6 5 4 3 2 1 0

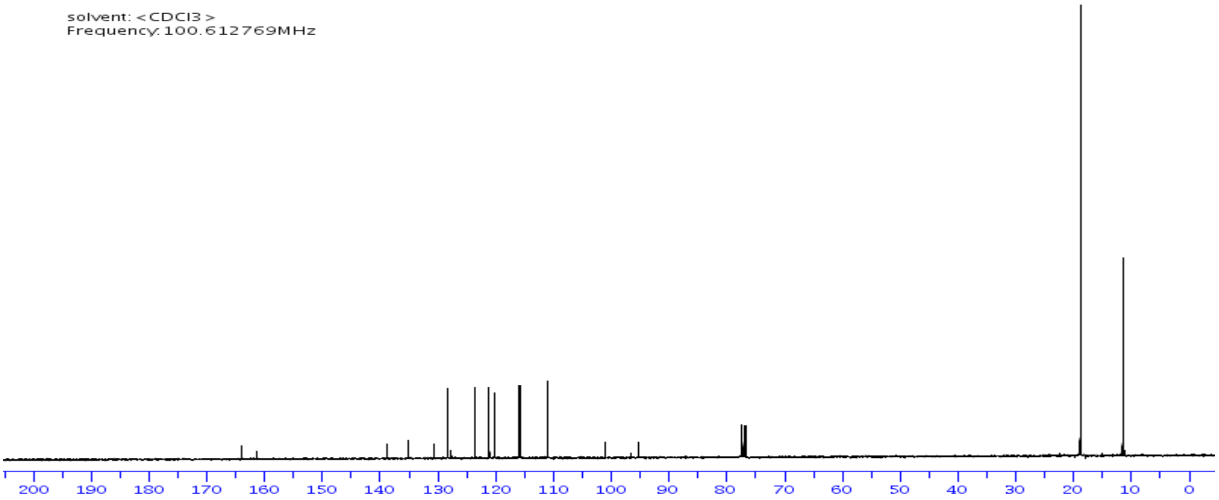
solvent: <CDCl3>
Frequency: 100.612769MHz
200 190 180 170 160 150 140 130 120 110 100 90 80 70 60 50 40 30 20 10 0

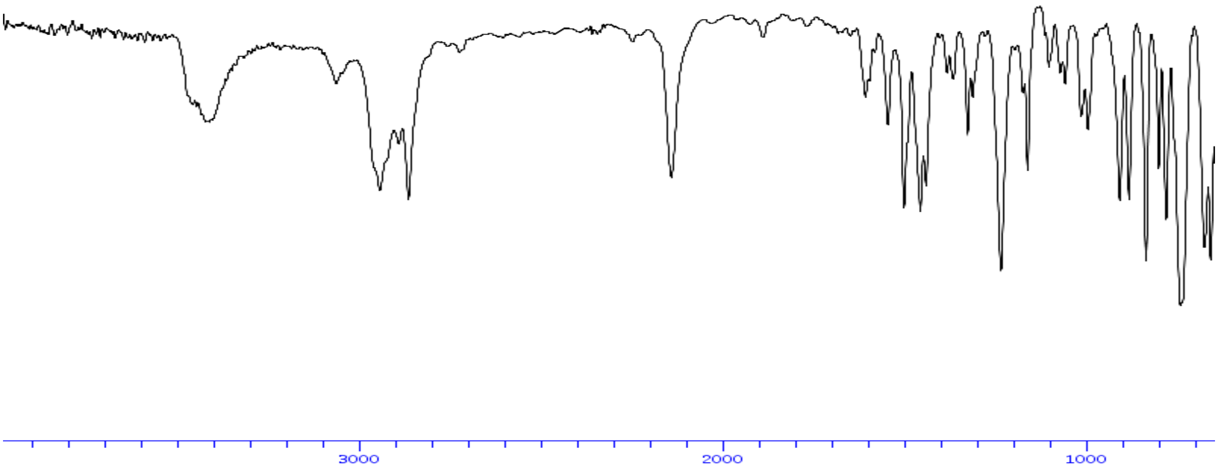
3000 2000 1000

solvent: <CDCl3>
Frequency: 400.13MHz

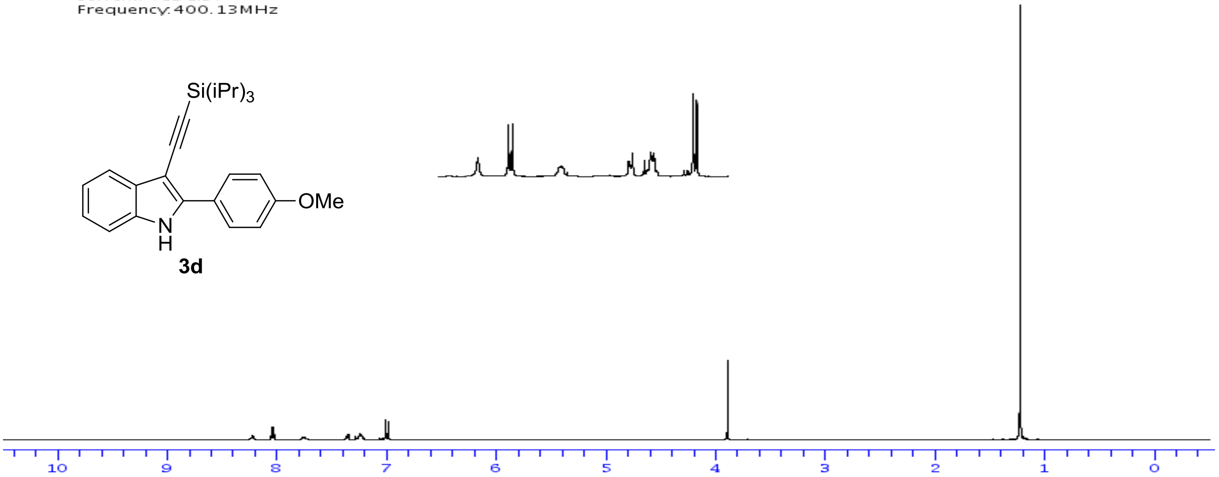


**3d**

solvent: <CDCl3>
Frequency: 100.612769MHz

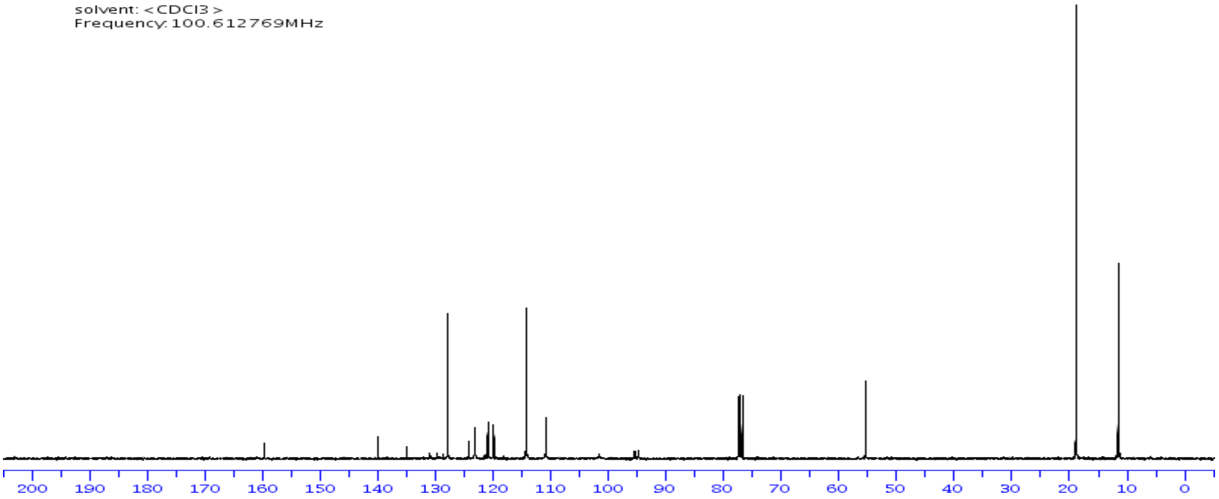


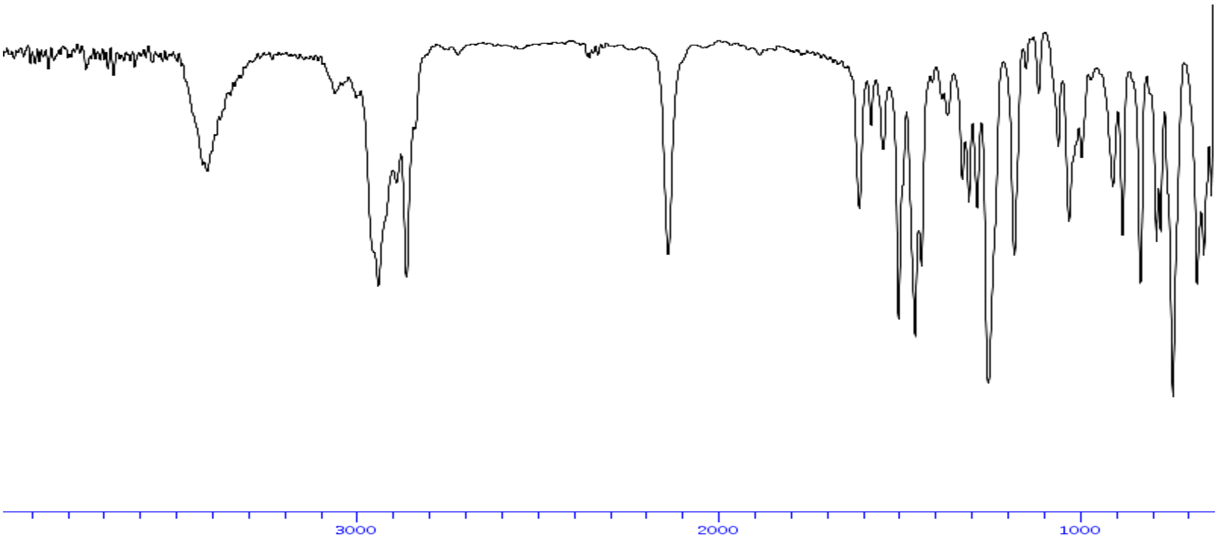

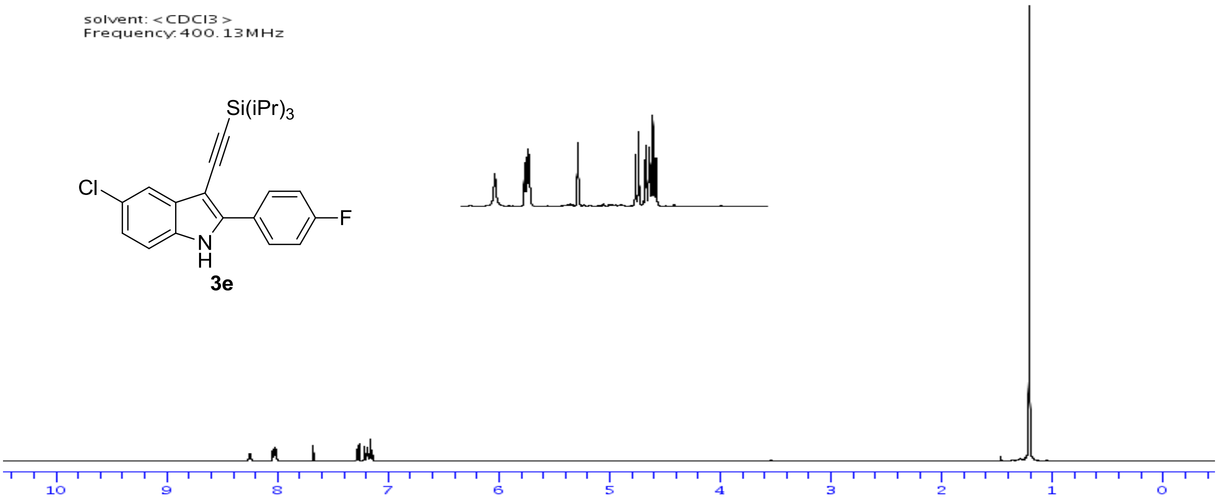
solvent: <CDCl3>
Frequency: 400.13MHz
Si(iPr)3
Cl
F
N
H
3e
10 9 8 7 6 5 4 3 2 1 0

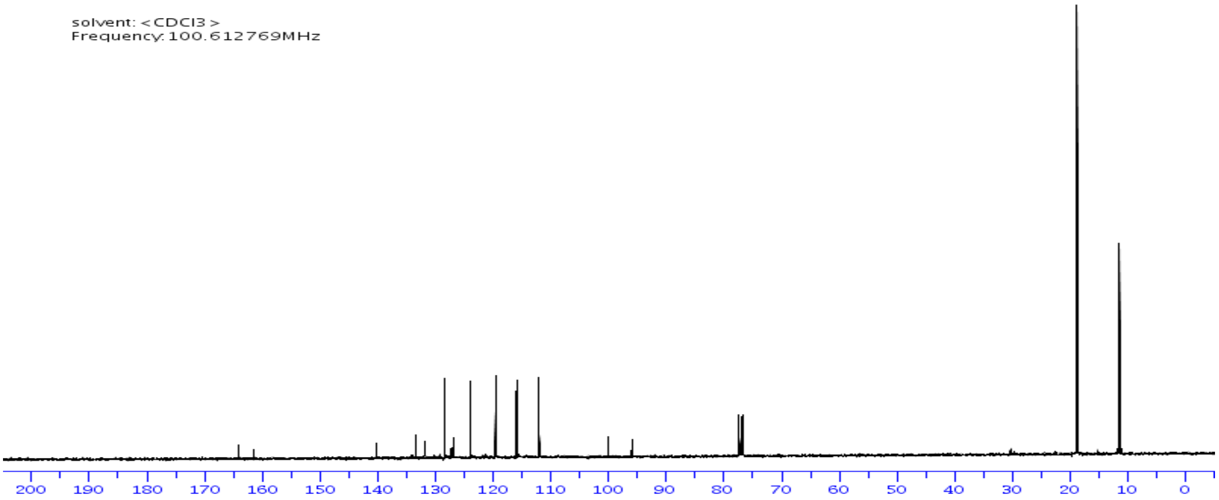
solvent: <CDCl3>
Frequency: 100.612769MHz
200 190 180 170 160 150 140 130 120 110 100 90 80 70 60 50 40 30 20 10 0

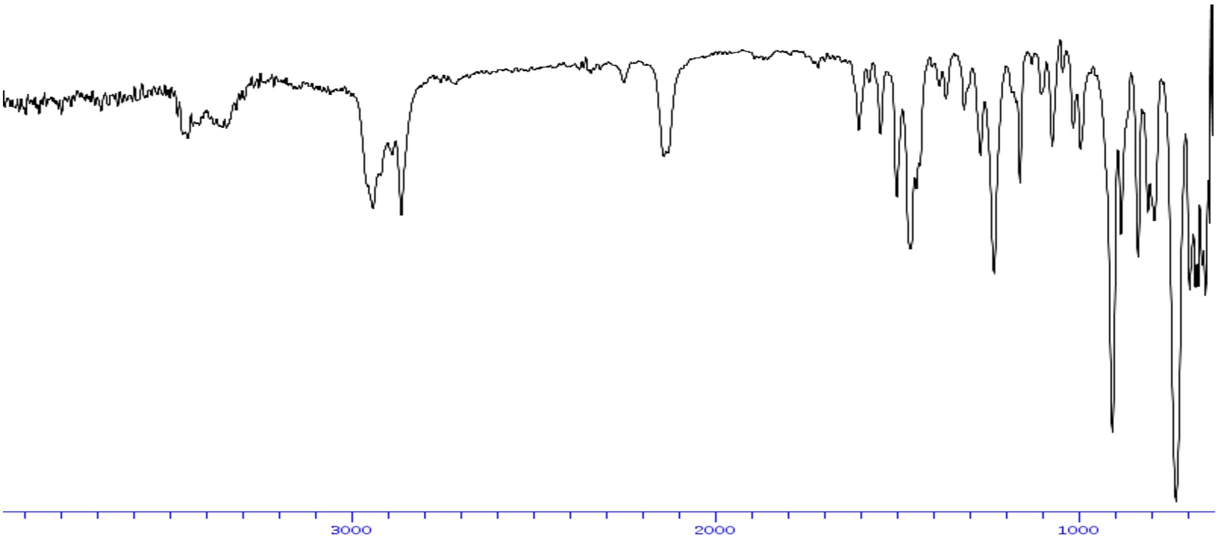
3000
2000
1000

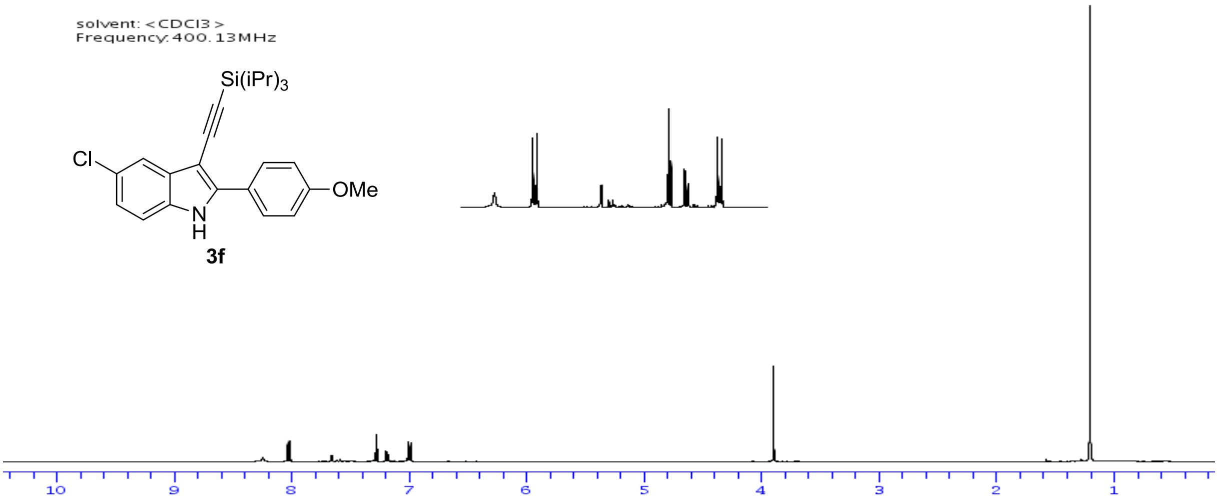
solvent: <CDCl3>
Frequency: 400.13MHz
Si(iPr)3
Cl
OMe
N
H
3f

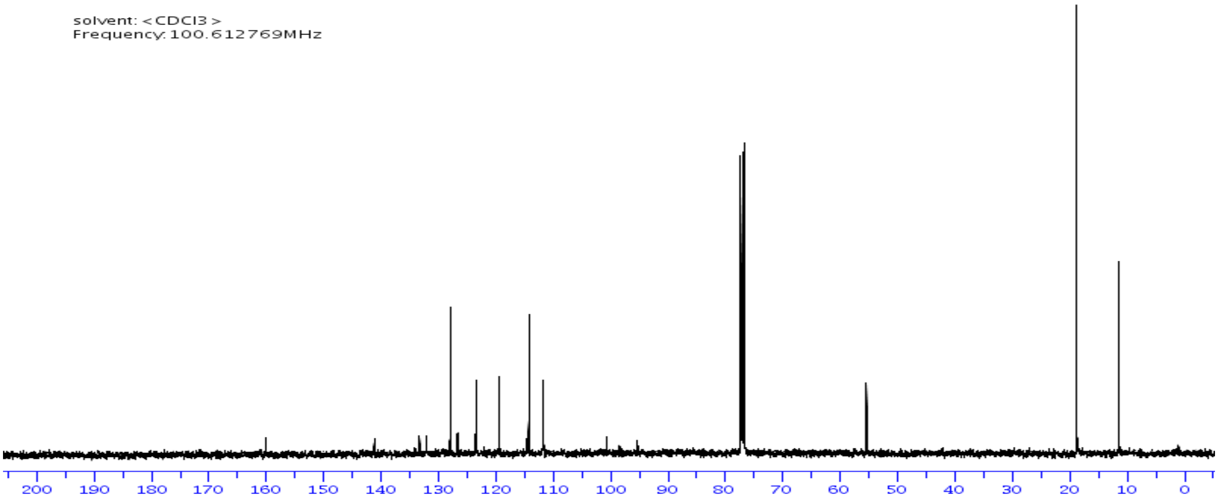
solvent: <CDCl3>
Frequency: 100.612769MHz

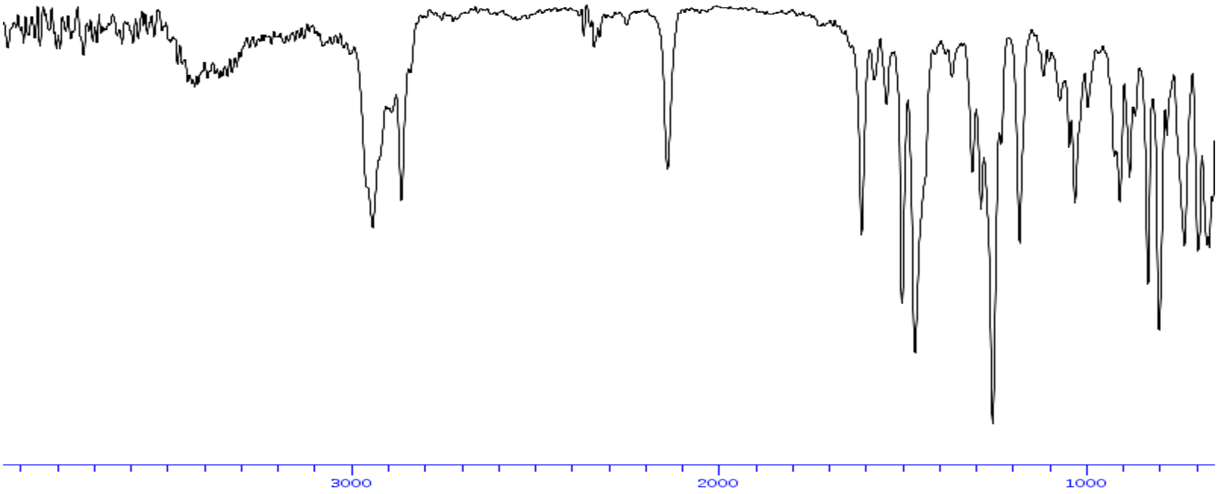

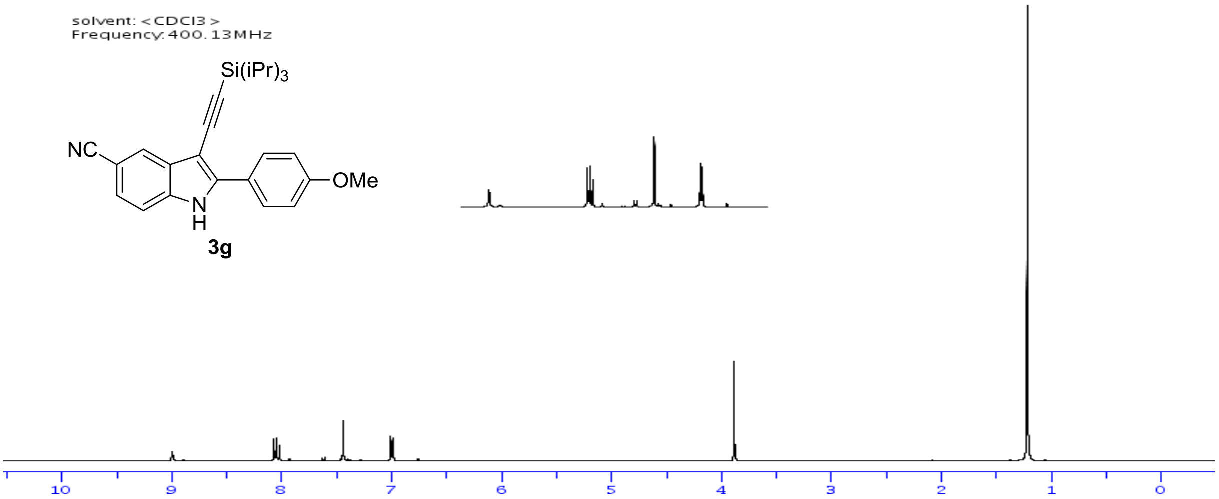
solvent: <CDCl3>
Frequency: 400.13MHz
Si(iPr)3
NC
OMe
N
H
3g

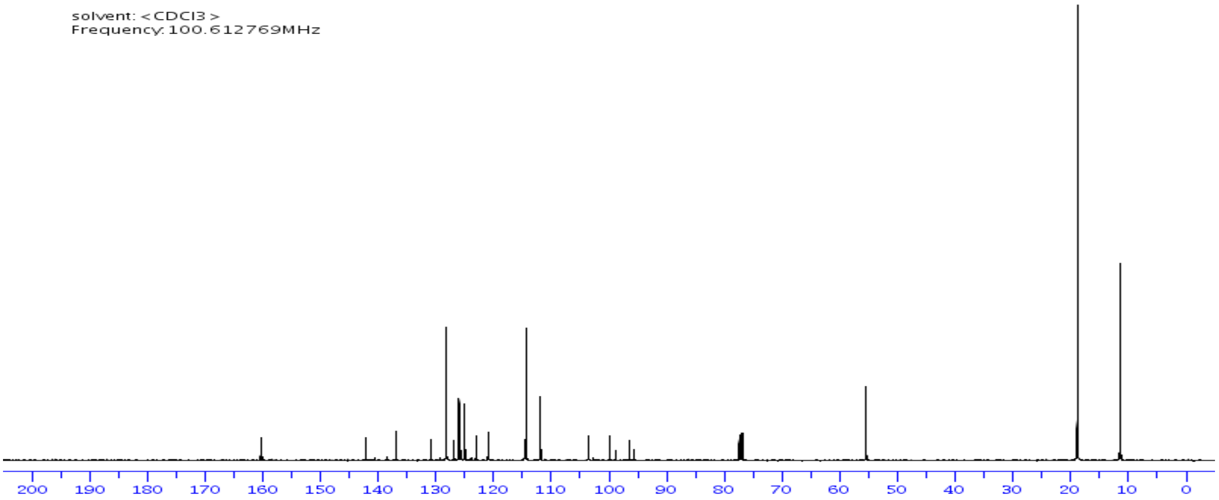
solvent: <CDCl3>
Frequency: 100.612769MHz

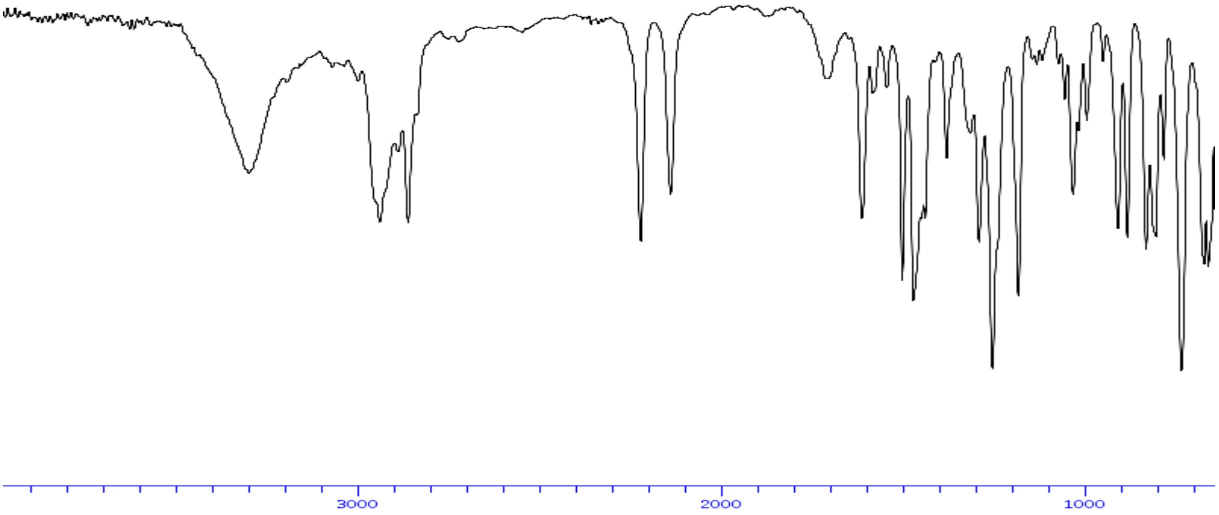

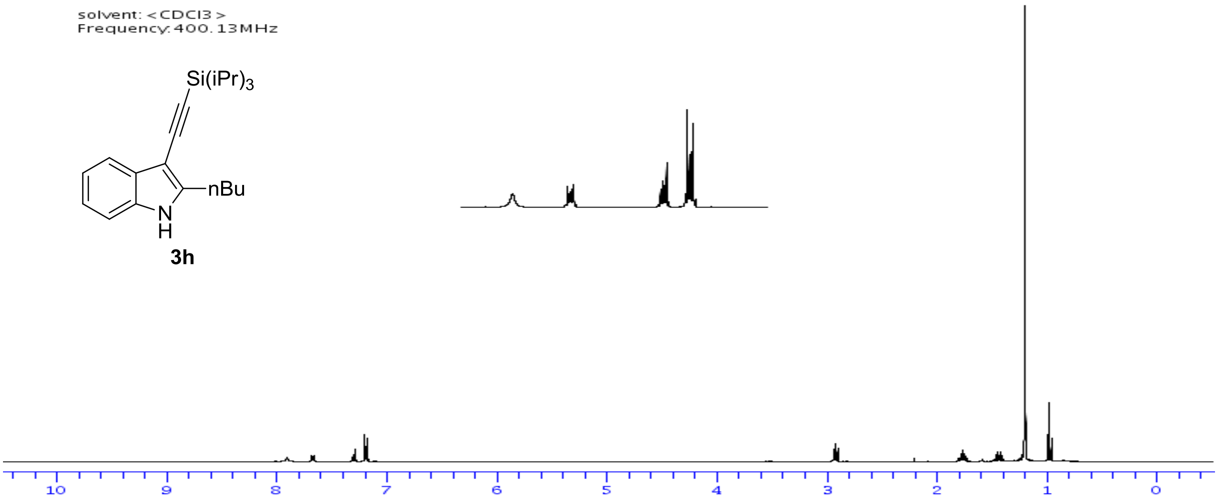
solvent: <CDCl3>
Frequency: 400.13MHz
Si(iPr)3
nBu
N
H
3h
10 9 8 7 6 5 4 3 2 1 0

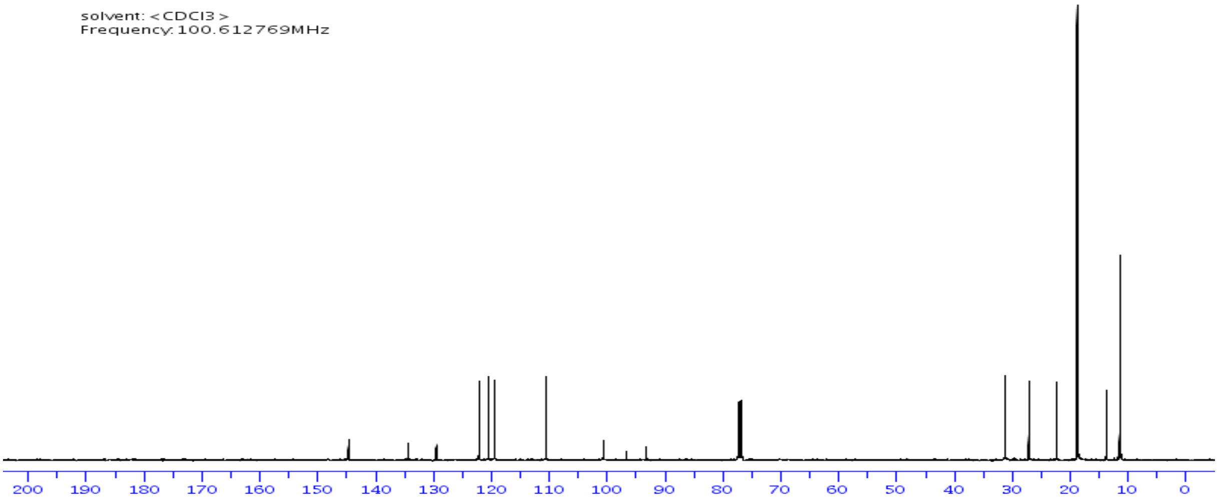
solvent: <CDCl3>
Frequency: 100.612769MHz
200 190 180 170 160 150 140 130 120 110 100 90 80 70 60 50 40 30 20 10 0

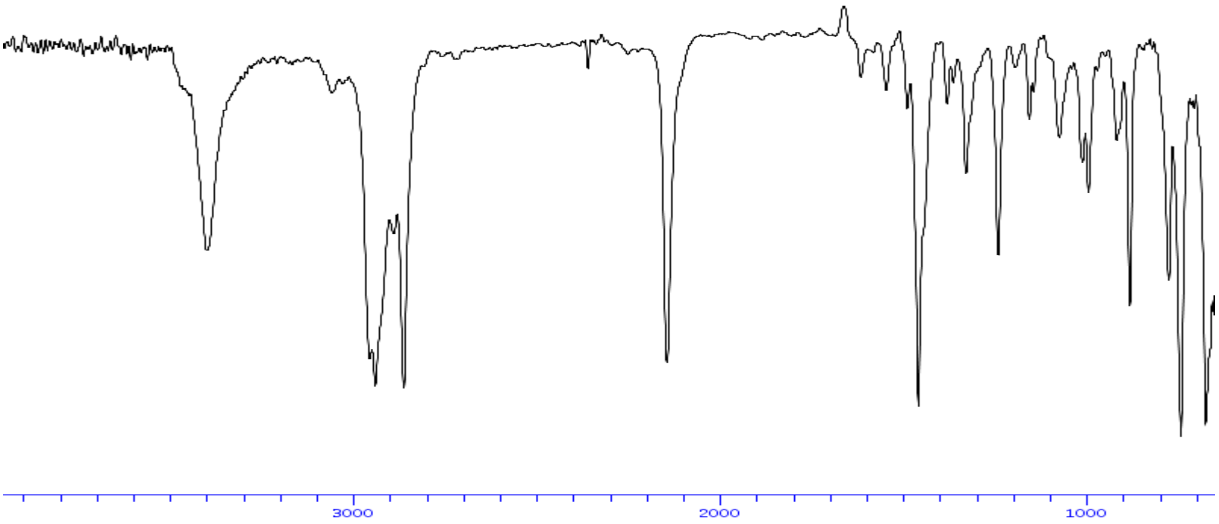
3000
2000
1000
